# Implementation interventions for musculoskeletal programs of care in the active military and barriers, facilitators, and outcomes of implementation: a scoping review

**DOI:** 10.1186/s13012-019-0931-1

**Published:** 2019-08-16

**Authors:** Carol Cancelliere, Deborah Sutton, Pierre Côté, Simon D. French, Anne Taylor-Vaisey, Silvano A. Mior

**Affiliations:** 1 Faculty of Health Sciences, University of Ontario Institute of Technology, 2000 Simcoe Street North, Science building, Room 3000, Oshawa, Ontario L1H 7K4 Canada; 2 UOIT-CMCC Centre for Disability Prevention and Rehabilitation, Faculty of Health Sciences, University of Ontario Institute of Technology (UOIT), 2000 Simcoe Street North, Science building, Room 3000, Oshawa, Ontario L1H 7K4 Canada; 3 UOIT-CMCC Centre for Disability Prevention and Rehabilitation, Canadian Memorial Chiropractic College (CMCC), 6100 Leslie Street, Toronto, Ontario M2H 3J1 Canada; 4 Division of Research and Innovation, Canadian Memorial Chiropractic College, 6100 Leslie Street, Toronto, Ontario M2H 3J1 Canada; 5 Canada Research Chair in Disability Prevention and Rehabilitation, Faculty of Health Sciences, University of Ontario Institute of Technology (UOIT), 2000 Simcoe Street North, Science building, Room 3000, Oshawa, Ontario L1H 7K4 Canada; 6 School of Rehabilitation Therapy, Faculty of Health Sciences, Queen’s University, Louise D. Acton Building, 31 George Street, Kingston, Ontario K7L 3N6 Canada; 7 Department of Chiropractic, Faculty of Science and Engineering, Macquarie University, Macquarie Park, NSW 2109 Australia

**Keywords:** Military personnel, Wounds and injuries, Therapeutics, Scoping review, Implementation science

## Abstract

**Background:**

Musculoskeletal disorders are common in the active military and are associated with significant lost duty days and disability. Implementing programs of care to manage musculoskeletal disorders can be challenging in complex healthcare systems such as in the military. Understanding how programs of care for musculoskeletal disorders have been implemented in the military and how they impact outcomes may help to inform future implementation interventions in this population.

**Methods:**

We conducted a scoping review using the modified Arksey and O’Malley framework to identify literature on (1) implementation interventions of musculoskeletal programs of care in the active military, (2) barriers and facilitators of implementation, and (3) implementation outcomes. We identified studies published in English by searching MEDLINE, CINAHL, Embase, and CENTRAL (Cochrane) from inception to 1 June 2018 and hand searched reference lists of relevant studies. We included empirical studies. We synthesized study results according to three taxonomies: the Effective Practice and Organization of Care (EPOC) taxonomy to classify the implementation interventions; the capability, opportunity, motivation-behavior (COM-B) system to classify barriers and facilitators of implementation; and Proctor et al.’s taxonomy (Adm Policy Ment Health 38:65–76, 2011) to classify outcomes in implementation research.

**Results:**

We identified 1785 studies and 16 were relevant. All but two of the relevant studies were conducted in the USA. Implementation interventions were primarily associated with delivery arrangements (e.g., multidisciplinary care). Most barriers or facilitators of implementation were environmental (physical or social). Service and client outcomes indicated improved efficiency of clinical care and improved function and symptomology. Studies reporting implementation outcomes indicated the programs were acceptable, appropriate, feasible, or sustainable.

**Conclusion:**

Identification of evidence-based approaches for the management of musculoskeletal disorders is a priority for active-duty military. Our findings can be used by military health services to inform implementation strategies for musculoskeletal programs of care. Further research is needed to better understand (1) the components of implementation interventions, (2) how to overcome barriers to implementation, and (3) how to measure implementation outcomes to improve quality of care and recovery from musculoskeletal disorders.

## Background

Musculoskeletal disorders are the most common reason military members seek health care, irrespective of the setting, whether deployed or at home base [1, 2]. These disorders are associated with lost productivity due to sick parade attendance and lost duty days [3–7] and are responsible for 42% of all medical releases in the Canadian Armed Forces [3]. Approximately 1.6 million musculoskeletal injuries occur annually within the US Department of Defense, which account for 25 million lost duty days [1]. Musculoskeletal disorders are a leading contributor of healthcare visits and costs in the US military, accounting for approximately 2.4 million medical visits and US$548 million in direct patient care costs [8]. Frequently reported musculoskeletal disorders by military personnel are of the lower limb, low back, neck, and shoulder [9–11]. These disorders are commonly caused by overuse, exacerbations of previous injuries, sports, physical training, lifting and carrying, and walking on uneven terrain [12]. Risk factors for musculoskeletal injury in the military include poor results in running and lifting tests, high waist circumference, high body mass index, previous musculoskeletal symptoms, poor school success, old age, higher enlisted rank, female sex, months deployed, and time spent standing [13, 14].

Efficient and effective strategies to manage musculoskeletal disorders in the active military are of great importance. Evidence-based treatments for musculoskeletal disorders include a focus on active versus passive treatment, structured education, exercise, and manual and cognitive behavioral therapies [15]. Strategies for implementing evidence-based practices should be tailored for specific settings and contexts [16], especially in complex systems. The military is a complex system with widely dispersed base locations that vary in size, human and facility resources, duties, and composition (e.g., full-time active duty, reserve, or National guard) [17]. Implementing services or programs of care is challenging in complex systems and is influenced by contextual factors such as the military culture, support of interest groups, chain of command, and resources. Therefore, careful consideration to implementation methods or interventions is important to facilitate uptake of evidenced-based programs of care.

Implementation interventions are methods or techniques designed to change behaviors at organizational, practitioner, or patient levels [18, 19] and to enhance the adoption of a clinical intervention [20]. The Cochrane Effective Practice and Organization of Care (EPOC) Group has categorized these interventions in a taxonomy of delivery arrangements, financial arrangements, governance arrangements, and implementation strategies [21]. Examples of implementation interventions include the development of multidisciplinary teams (delivery arrangements), the use of financial incentives for health professionals and organizations (financial arrangements), policies that regulate what health professionals can do (governance arrangements), and educational meetings and clinical practice guidelines (implementation strategies). Implementation interventions may be tailored to overcome barriers to implementation, using a framework such as the Behavior Change Wheel [22]. In this approach, barriers are classified using the capability, opportunity, motivation-behavior (COM-B) system and mapped onto specific interventions designed to overcome implementation barriers. Finally, appropriate outcomes are necessary to evaluate the success of healthcare interventions. Proctor et al.’s taxonomy of outcomes in implementation research distinguishes implementation outcomes, which are the effects of deliberate and purposive actions to implement new treatments, practices, and services [23], from service and client outcomes, which are usually reported on in scientific papers rather than implementation outcomes. However, it is important to report on implementation outcomes because they are key intermediate outcomes in relation to service system or clinical outcomes in treatment effectiveness and quality of care research [23, 24]. Clearly, if interventions are to result in desired changes in clinical or service outcomes, they need to be implemented well [23].

Understanding the implementation of interventions—in particular *how* programs of care are implemented to manage musculoskeletal disorders—may inform the overall management of these disorders in active military populations, beyond the specific clinical intervention. This may ultimately help to improve patient outcomes and cost-effectiveness of care to benefit military members and the system. To the best of our knowledge, there are no previous studies synthesizing implementation interventions of musculoskeletal programs of care in this population, barriers and facilitators to implementation, or outcomes. Therefore, the purpose of this scoping review was to describe (1) implementation interventions used to deliver programs of care, (2) barriers or facilitators of implementation, and (3) outcomes of implementation used in the management of musculoskeletal disorders in the active military.

## Methods

We used the modified “Arksey and O’Malley framework” to examine the extent, range, and nature of the research activity related to the implementation of musculoskeletal programs of care for military personnel [25–27]. This approach involves six stages: (1) identifying the research question; (2) defining the scope of the review; (3) study selection; (4) charting the data; (5) collating, summarizing, and reporting the results; and (6) stakeholder consultation.

### Stage 1: Identifying the research question

Our scoping review was guided by the following research questions: “What implementation interventions have been used to deliver programs of care for managing musculoskeletal disorders among active military personnel?” and “What were the barriers, facilitators, and outcomes of implementation?”

### Stage 2: Defining the scope of the review

We defined eligibility criteria a priori. Studies were included if they met the following criteria to explore implementation interventions, barriers and facilitators of implementation, or outcomes of implementation:

1. *Population*: participants were active military personnel (including reservist and National Guard).
2. *Program of care or intervention*: designed for the clinical management of musculoskeletal disorders.
3. *Study design*: any primary empirical study (e.g., quantitative, qualitative, or mixed methods) published in the peer-reviewed literature.
4. *Implementation intervention*: any technique or method used to implement an evidence-based musculoskeletal program of care or intervention.
5. *Barriers and facilitators*: any factor that either impeded or enabled the implementation of an evidence-based musculoskeletal program of care or intervention.
6. *Outcomes*: implementation outcomes (acceptability, adoption, appropriateness, costs, feasibility, fidelity, penetration, or sustainability); service outcomes (efficiency, safety, effectiveness, equity, patient-centeredness, or timeliness); or patient outcomes (satisfaction, function, or symptomatology) [23].
7. *Language of publication*: studies published in the English language.

Studies were excluded if (1) participants were non-active duty military personnel, e.g., veterans; (2) programs of care were designed for non-musculoskeletal disorders, serious injuries, or pathologies; and (3) they were in the gray literature, e.g., theses, newsletters, and informal communication.

### Stage 3: Study selection

We developed our search strategy in consultation with a health sciences librarian (Appendix). A second librarian reviewed the search using the Peer Review of Electronic Search Strategies (PRESS) checklist [28]. The following electronic databases were searched from database inception to June 1, 2018: MEDLINE (Ovid®), CINAHL (EBSCO), Embase (Ovid®), and Cochrane Central Register of Controlled Trials through Ovid® (CENTRAL). The search terms included subject headings specific to each database and free-text terms relevant to musculoskeletal disorders, the military, and implementation interventions (Appendix). We reviewed the reference lists of all eligible studies for additional studies not identified from the electronic database search. Databases containing the results of the searches were created using EndNote X6. We used the Preferred Reporting Items for Systematic Reviews and Meta-analyses (PRISMA) extension for scoping reviews (PRISMA-ScR) flow chart to track the number of studies at each stage of the review.

Two researchers screened studies using a two-step screening process. In phase I, two reviewers (CC, DS) independently screened titles and abstracts to determine eligibility. They classified studies as relevant, possibly relevant, or irrelevant. In phase II, the reviewers independently reviewed manuscripts of possibly relevant studies to make a final determination of eligibility. The reviewers met to resolve disagreements and reach consensus in both steps. A third independent reviewer was available to discuss and resolve disagreements.

### Stage 4: Charting the data

Two reviewers (CC, DS) independently charted and coded the following data from eligible studies: (1) author and year of publication; (2) study design; (3) clinical setting and participant characteristics; (4) program of care and implementation intervention according to the EPOC taxonomy [21]; (5) barriers and facilitators to implementation according to the COM-B system [22]; and (6) outcomes (implementation, service, patient) according to Proctor et al.’s taxonomy of outcomes for implementation research [23].

### Stage 5: Analysis

We analyzed the data using a descriptive numerical summary, a qualitative thematic analysis, and by applying meaning to our results [25–27].

#### Descriptive numerical summary

We described the characteristics of included studies, such as the number of studies included, types of study design, characteristics of the study populations, types of musculoskeletal disorders, and countries where the studies were conducted.

#### Qualitative thematic analysis

We categorized the data into three sections: implementation interventions of the musculoskeletal programs of care (according to EPOC [21]), barriers and facilitators of implementation (according to COM-B [22]), and the implementation research outcomes taxonomy by Proctor et al. [23].

The EPOC taxonomy includes four domains of health systems interventions: (1) delivery arrangements: changes in how, when, and where health care is organized and delivered, and who delivers health care; (2) financial arrangements: changes in how funds are collected, insurance schemes, how services are purchased, and the use of targeted financial incentives or disincentives; (3) governance arrangements: rules or processes that affect the way in which powers are exercised, particularly with regard to authority, accountability, openness, participation, and coherence; and (4) implementation strategies: interventions designed to bring about changes in healthcare organizations, the behavior of healthcare professionals, or the use of health services by healthcare recipients.

The COM-B framework is useful for understanding behavior and behavior change [22]. *Capability* is defined as the individual’s *psychological* and *physic*al capacity to engage in the desired activity. It includes having the necessary knowledge and skills, such as the knowledge to diagnose a patient with a lumbar disc herniation (psychological capacity), and the skill to take a blood sample (physical capacity). *Opportunity* is defined as all the factors that lie outside the individual, either in the *physical* or *social* environment, that make the behavior possible or prompt it. An example of a physical opportunity is being able to go running because you own running shoes. A social opportunity (or a lack thereof) is being able to dress casually at home but not at a business meeting. *Motivation* is defined as all the brain processes that energize and direct behavior, including goals, conscious and analytical decision-making, habitual processes, and emotional responding. Motivation can be *reflective*, involving evaluations and plans, such as deciding to buy a car based on its safety features. On the other hand, *automatic* motivation involves emotional and impulsive processes such as deciding to buy a car based on its attractiveness in an advertising campaign.

Finally, Proctor et al. [23] have classified the outcomes of interventions, services, or innovations as (1) *implementation outcomes* (acceptability, adoption, appropriateness, costs, feasibility, fidelity, penetration, and sustainability), (2) *service outcomes* (efficiency, safety, effectiveness, equity, patient-centeredness, and timeliness), and (3) *client outcomes* (symptomatology, function, and satisfaction) [23]. They defined the implementation outcomes as follows. *Acceptability* is the perception among implementation stakeholders that a given intervention is agreeable, palatable, or satisfactory in terms of its content, complexity, or comfort. *Appropriateness* is the perceived fit, relevance, or compatibility of the intervention for a given practice setting, provider, or consumer; or the perceived fit of the intervention to address a particular problem. *Adoption* or “uptake” is defined as the intention, initial decision, or action to try or employ an intervention. The *cost* impact of an implementation effort depends upon the costs of the particular intervention, the implementation strategy used, and the location of service delivery. *Feasibility* is defined as the extent to which an intervention can be successfully used or carried out within a given setting. *Fidelity* is defined as the degree to which an intervention was implemented as was intended. *Penetration* is defined as the integration of a practice with a service setting and its subsystems (i.e., an intervention’s institutionalization). *Sustainability* is defined as the *extent* to which a newly implemented intervention is maintained or institutionalized within a setting.

#### Applying meaning to results

We considered the meaning of our scoping study results and the broader implications for research, policy, and practice.

### Stage 6: Consultation

We consulted with available authors of the studies included in our review for the purpose of elaborating on their key findings. We also consulted with organizations (Canadian Armed Forces and the Canadian and Ontario Chiropractic Associations) and other experts during a summer institute (Knowledge Translation Canada, June 2017) for their perspectives and experiences regarding barriers and facilitators to health program implementation. We did not conduct a formal qualitative content analysis of comments from the authors, organizational representatives, or experts.

## Results

### Description of included studies

**Fig. 1:**
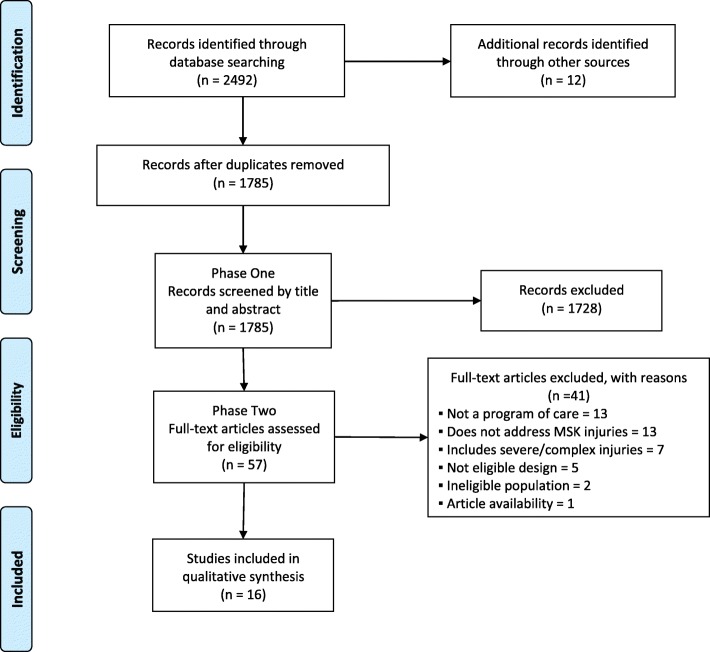
Preferred reporting items for scoping review (PRISMA-ScR)

The study selection process is shown in Fig. 1. After excluding duplicates, the electronic database search and reference list search of eligible studies produced 1785 studies. Fifty-seven studies underwent phase II full-text screening. Sixteen relevant studies [29–44] were identified (eight observational studies including case reports and series, cross-sectional and non-experimental studies, and cohorts; four mixed methods studies; three pilot studies; one qualitative study). Fourteen studies were conducted in the US, and one study each was conducted in Canada and Sweden. Low back pain [29, 31–35, 37, 40, 42] was the most common musculoskeletal disorder targeted, followed by musculoskeletal disorders as a group including spinal pain [30, 38, 39, 41, 43, 44] and neck pain [36].

We synthesized the study results according to the implementation interventions of the musculoskeletal programs of care used as per the EPOC taxonomy (Table 1, 16 studies), barriers and facilitators of implementation using the COM-B system (Table 2, 14 studies), and outcomes (Table 3, 16 studies).

**Table 1:** Implementation interventions of musculoskeletal programs of care for active military classified using the Effective Practice and Organization of Care (EPOC) taxonomy

Implementation interventions according to Effective Practice and Organization of Care (EPOC) taxonomy		Author(s), year, study design	Clinical setting/participants type and/or number (*n*)	Description of Implementation Interventions and Programs of Care
Delivery arrangements				
How and when care is delivered	Coordination of care among different providers	Green et al., 2006 [35], case report	Military treatment facility, US US Marine Corps F/A-18 aviation instructor, age 36	Interdisciplinary management of low back pain Flight surgeon coordinated clinical consults with neurosurgeon, hospital physiatrist, physical therapist Physical therapist discussed case with chiropractor located in the same clinic
		Kelly et al., 1997 [39], qualitative case study	Recruit Training Command Great Lakes, US 1992: *n* = 292 1993: *n* = 246 1994: *n* = 529 1995: *n* = 898	Interdisciplinary management of musculoskeletal disorders Musculoskeletal team 3-tier approach: evaluate and diagnose injured recruit, and initiate appropriate level of treatment based on injury severity Physical therapist provided 5 days/week Well-established team protocols and an understanding of the particular injuries benefitting from physical therapist, and when to refer to physical therapist
		Lillie, 2010 [40], case report	Military treatment facility, US US Navy Petty Officer, age 40	Interdisciplinary management of low back pain Primary care, chiropractic physician, and orthopedic specialist provided coordinated care in an established multidisciplinary health system
		Rhon et al., 2017 [41], cross-sectional	Madigan Army Medical Centre, US National Guard, 116th Cavalry Brigade Combat Team *N* = 284 Average age 32 years	Interdisciplinary Reverse Soldier Readiness Program, Musculoskeletal Soldier Readiness Program Clinical Pathway Patients with multiple complaints, both musculoskeletal and non-musculoskeletal disorders, were referred to primary care for care coordination
		Ziemke et al., 2015 [42], quasi-experimental	Naval Medical Center Portsmouth, US Naval Medical Center San Diego, US US Navy and US Marine Corps service members, aged 18–64, seeking care for a work-disabling spine condition (2007–2009) *n* = 667	Interdisciplinary management of work-disabling nonspecific low back pain Service members referred to the Spine Team, where an orthopedic spine surgeon screens for a surgical or non-surgical consultation Non-surgical cases are distributed among the remaining members of the Spine Team (physical therapist, physician assistant, physiatrist, chiropractor) Referral by any team member to psychologist
Where care is provided and changes to the healthcare environment	Site of service delivery	Boudreau et al., 2006 [29], pilot	Outpatient department, Archie McCallum Hospital, Canadian Forces Base Stadacona, Canada Chiropractor (*n* = 2) MD (*n* = 12) Consecutive active military members with low back pain (*n* = 102)	Interdisciplinary management of musculoskeletal disorders On-site, outpatient treatment at military hospital for musculoskeletal disorders
		Green et al., 2006 [35], case report	Military treatment facility, US US Marine Corps F/A-18 aviation instructor, age 36	Interdisciplinary management of low back pain Chiropractor and physical therapist located in the same clinic
		James et al., 1981 [38], mixed methods	US army hospital, US Army Health Services Command data Physical therapists (*n* = 5) Active duty military (*n* = 3291)	Expanded physical therapist role as primary screener of musculoskeletal conditions Musculoskeletal evaluation clinic operates in conjunction with the regular physical therapist clinic
		Kelly et al., 1997 [39], qualitative case study	Recruit Training Command Great Lakes, US 1992: *n* = 292 1993: *n* = 246 1994: *n* = 529 1995: *n* = 898	Interdisciplinary management of musculoskeletal disorders Musculoskeletal team of physician, physical therapists, podiatrists, physician assistants, independent duty corpsmen, physical therapist technicians Share expertise in diagnosis and treatment of musculoskeletal injuries Training room created within the recruit medical clinic, and musculoskeletal team worked in collaboration with the Recruit Rehabilitation Unit (RRU) and the Recruit Convalescent Unit (RCU)
		Lillie, 2010 [40], case report	Military treatment facility, US US Navy Petty Officer, age 40	Interdisciplinary management of low back pain Primary care manager co-located with patient allowed for monitoring of progress and coordination of care
		McGee et al., 2017 [43], mixed methods	Moody Air Force Base, US 23rd Medical Group (outpatient clinic) (*n* = 12): physicians, physician assistants, nurse practitioners, physical therapists	“Physical Therapy First” orthopedic performance improvement initiative designed within Consolidated Framework for Implementation Research model Improve appropriate referrals and decrease inappropriate resource utilization for musculoskeletal injuries Orthopedic care provided through a private managed care network, changed by having consults further screened to allow for specialty care at US Navy Jacksonville Orthopedic Department (local to encourage collaboration between programs within a specific region)
	Environment	Brawley et al., 2012 [30], historical cohort	Marine Corps Base Camp Lejeunce Mainside and Camp Geiger, US Active duty service member placed on limited duty for primary upper or lower extremity injury (*n* = 8299)	Sports Medicine and Reconditioning Team (SMART) clinic model replacing traditional problem-based clinic model Athletic training room model with an open-bay configuration allowing for coordinated multidisciplinary approach, direct transition of care, communication between team members
		Kelly et al., 1997 [39], qualitative case study	Recruit Training Command Great Lakes, US 1992: *n* = 292 1993: *n* = 246 1994: *n* = 529 1995: *n* = 898	Interdisciplinary management of musculoskeletal disorders Training room format based on college athletic training room model
Who provides care and how the healthcare workforce is managed	Role expansion	James et al., 1975 [37], mixed methods	Non-teaching army hospital, US Physical therapist s (*n* = 8) Baseline phase (*n* = 950) Screening phase (*n* = 2296)	Expanded physical therapist role as primary screener for low back pain complaint using a decision guide Physical therapist role: evaluate patient, request x-rays, determine patient care, refer to physician for further evaluation
Coordination of care and management of care processes	Care pathways	Larsson et al., 2012 [44], non-experimental	Swedish Armed Forces who started military training: 2003: *n* = 120 (artillery) 2004: *n* = 356 (Ranger) 2004: *n* = 407 (engineer)	Musculoskeletal Screening Protocol: questionnaire (lifestyle factors, self-rated health) and physical tests for musculoskeletal complaints and functional limitations within first week of soldiers’ arrival Provided early rehabilitation or physical training/exercise programs
		McGee et al., 2017 [43], mixed methods	Moody Air Force Base, US Moody Air Force Base, US 23rd Medical Group (outpatient clinic) (*n* = 12): physicians, physician assistants, nurse practitioners, physical therapists	“Physical Therapy First” orthopedic performance improvement initiative designed within Consolidated Framework for Implementation Research model -Improve appropriate referrals and decrease inappropriate resource utilization for musculoskeletal injuries physical therapy was the first line of care and conservative treatment was exhausted before orthopedic specialty referral
	Communication between providers	Boudreau et al., 2006 [29], pilot study	Outpatient department, Archie McCallum Hospital, Canadian Forces Base Stadacona, Canada Chiropractor (*n* = 2) MD (*n* = 12) Consecutive active military members with low back pain (*n* = 102)	Interdisciplinary management of musculoskeletal disorders Initial report (examination findings, clinical impression, treatment plan, prognosis) Progress update after 10 treatments to MD for approval for further care
		Lillie 2010 [40], case report	Military treatment facility, US US Navy Petty Officer, age 40	Interdisciplinary management of low back pain Encourage weekly meetings with specialty providers
	Packages of care	Goertz et al., 2013 [34], pilot RCT study	William Beaumont Army Medical Center (WBAMC), US US active-duty military personnel, age 18–35, low back pain < 4 weeks duration Chiropractic manipulative therapy, *n* = 45 Standard medical care, *n* = 46	Interdisciplinary management of low back pain Chiropractic manipulative therapy, 2/week for 4 weeks High velocity low amplitude manipulation, plus brief massage, ice/heat lumbar region, stretching or McKenzie exercises, advice on activites of daily living, postural/ergonomic advice, mobilization, with standard medical care Standard Medical Care: history and physical exam, diagnostic imaging as indicated, self-management education including activity as tolerated, pharmacological management (analgesics, anti-inflammatory agents), physical therapy, modalities, e.g., heat/ice, referral to pain clinic
		Green et al., 2006 [35], case report	Military treatment facility, US US Marine Corps F/A-18 aviation instructor, age 36	Interdisciplinary management of low back pain Chiropractor informed flight surgeon of the course of care Chiropractor and physical therapist discussed case to ensure care was complimentary and not redundant
	Referral systems	Boudreau et al., 2006 [29], pilot study	Outpatient department, Archie McCallum Hospital, Canadian Forces Base Stadacona, Canada Chiropractor (*n* = 2) MD (*n* = 12) Consecutive active military members with low back pain (*n* = 102)	Interdisciplinary management of musculoskeletal disorders Referral required by general practitioner or medical specialist to access chiropractor
		Green et al., 2006 [35], case report	Military treatment facility, US US Marine Corps F/A-18 aviation instructor, age 36	Interdisciplinary management of low back pain Flight surgeon ordered consults with neurosurgeon, hospital physiatrist, and physical therapist
		Green et al., 2010 [36], case report	Naval hospital, US US Marine Corps F/A-18 aviation instructor, age 38	Interdisciplinary management of neck pain Flight surgeon referral to on-station chiropractor
		James et al., 1975 [37], mixed methods	Non-teaching army hospital, US Physical therapists (*n* = 8) Baseline phase (*n* = 950) Screening phase (*n* = 2296)	Expanded role as primary screener for low back pain complaint using a decision guide Referral to physical therapist by physician or non-physician health care worker (e.g., army corpsman, nurse clinician, physicians’ assistant)
		James et al., 1981 [38], mixed methods	US army hospital, US Army Health Services Command data Physical therapists (*n* = 5) Active duty military (*n* = 3291)	Expanded physical therapist role as primary screener musculoskeletal conditions Musculoskeletal evaluation clinic Patients assigned in order, with other patients to physical therapists in expanded musculoskeletal role
		Lillie, 2010 [40], case report	Military treatment facility, US US Navy Petty Officer, age 40	Interdisciplinary management of low back pain Primary care manager (naval flight surgeon) manages and coordinates care of each patient Referral to orthopedic specialist and chiropractor
		McGee et al., 2017 [43], mixed methods	Moody Air Force Base, US 23rd Medical Group (outpatient clinic) (*n* = 12): physicians, physician assistants, nurse practitioners, physical therapists	“Physical Therapy First” orthopedic performance improvement initiative designed within Consolidated Framework for Implementation Research model Improve appropriate referrals and decrease inappropriate resource utilization for musculoskeletal injuries Physical Therapist Director acted as gatekeeper for all musculoskeletal consults requested by primary care staff Resource Management Officer consolidated all active duty orthopedic consults daily coordinating care between military treatment facility and civilian network
		Rhon et al., 2017 [41], cross-sectional	Madigan Army Medical Centre, US National Guard, 116th Cavalry Brigade Combat Team *N* = 284 Average age 32 years	Interdisciplinary Reverse Soldier Readiness Program, Musculoskeletal Soldier Readiness Program Clinical Pathway Soldier Readiness Program process occurred during one day in which the medical screener referred patients with primarily musculoskeletal injuries to Musculoskeletal Soldier Readiness Program Clinical Pathway which included physical therapist, physiatrist, and sports medicine physician. Care provided within 72 h Musculoskeletal Soldier Readiness Program Clinical Team gatekeepers to orthopedic surgeons, podiatry, and occupational therapist
		Ziemke et al., 2015 [42], quasi-experimental	Naval Medical Center Portsmouth, US Naval Medical Center San Diego, US US Navy and US Marine Corps service members, aged 18–64, seeking care for a work-disabling spine condition (2007-2009) *n* = 667	Interdisciplinary management of work-disabling nonspecific low back pain Service members are referred to the Spine Team Orthopedic spine surgeon screens for a surgical or non-surgical consultation Non-surgical cases are distributed among the remaining members of the Spine Team (physical therapist, physician assistant, physiatrist, chiropractor) Referral by any team member to psychologist
	Teams	Boudreau et al., 2006 [29], pilot study	Outpatient department, Archie McCallum Hospital, Canadian Forces Base Stadacona, Canada Chiropractor (*n* = 2) MD (*n* = 12) Consecutive active military members with low back pain (*n* = 102)	Interdisciplinary management of musculoskeletal disorders Individual chiropractors encouraged to work with other hospital departments on shared patients
		Campello et al., 2012 [31], pilot RCT study	Naval Medical Center, Portsmouth, Virginia, US Active duty service members presenting for low back pain at Sewell’s Point Branch Medical Clinic (*n* = 33)	Multidisciplinary ‘Backs to Work’ program compared to current standard care “Backs to Work” coordinated multidisciplinary, reconditioning program by physical therapist, MD and psychologist. Graded, goal-oriented active physical reconditioning program that includes aerobic conditioning, strength training, flexibility exercise, cognitive behavioural therapy (education about how psychosocial variables affect pain, relaxation training, modification of maladaptive beliefs, problem solving) Care providers worked as a team led by a clinical coordinator, who was responsible for coordination of care and communication among all healthcare providers and the service members command and/or workplace
		Kelly et al., 1997 [39], qualitative case study	Recruit Training Command Great Lakes, US 1992: *n* = 292 1993: *n* = 246 1994: *n* = 529 1995: *n* = 898	Interdisciplinary management of musculoskeletal disorders Environment of systematic collaboration Formal weekly team meetings to discuss progress Continual education and “curb-side” consults with physical therapist allowed for proper prescription of physical therapy treatment
		Larsson et al., 2012 [44], non-experimental	Swedish Armed Forces who started military training: 2003: *n* = 120 (artillery) 2004: *n* = 356 (ranger) 2004: *n* = 407 (engineer)	Musculoskeletal Screening Protocol Enhanced teamwork between officers and unit physiotherapists to give more awareness of early problems and adjust soldiers’ load Testing was led by physiotherapist; officers registered results of the testing
		Lillie, 2010 [40], case report	Military treatment facility, US US Navy Petty Officer, age 40	Interdisciplinary management of low back pain Primary care manager, chiropractor, orthopedic specialist (military and civilian)
		Rhon et al., 2017 [41], cross-sectional	Madigan Army Medical Centre, US National Guard, 116th Cavalry Brigade Combat Team *N* = 284 Average age 32 years	Interdisciplinary Reverse Soldier Readiness Program, Musculoskeletal Soldier Readiness Program Clinical Pathway Musculoskeletal Soldier Readiness Program Clinical Pathway team which included physical therapist, physiatrist and sports medicine physician. Musculoskeletal Soldier Readiness Program Clinical Team gatekeepers to orthopedic surgeons, podiatry and occupational therapist (not co-located with Musculoskeletal Soldier Readiness Program Clinical Pathway team)
		Ziemke et al., 2015 [42], quasi-experimental	Naval Medical Center Portsmouth, US Naval Medical Center San Diego, US US Navy and US Marine Corps service members, aged 18–64, seeking care for a work-disabling spine condition (2007–2009) *n* = 667	Interdisciplinary management of work-disabling nonspecific low back pain Spine team: 2 orthopedic spine surgeons; 1–2 orthopedic physical therapists (1 specialized training in spine), clinical psychologist with specialized training in pain management, physician, physiatrist
Information and communication technology (ICT)		Lillie, 2010 [40], case report	Military treatment facility, US US Navy Petty Officer, age 40	Interdisciplinary management of low back pain Electronic health record has built in referral process to facilitate referral, e.g., to chiropractor Electronic health record accessible to all military providers
Implementation strategies				
Interventions targeted at healthcare organizations	Organizational culture	Feuerstein et al., 2006 [33], cross-sectional	Military health service healthcare services within the continental US, fiscal years 1998–2002 Military health service beneficiaries, age 18–65, who completed a Health Care Survey of Department of Defense Beneficiaries (HCSDB)	Implementation of clinical practice guideline for the diagnosis and management of acute low back pain Establish leadership support Handbook to guide adoption of low back pain clinical practice guideline within Military Health Service
		McGee et al., 2017 [43], mixed methods	Moody Air Force Base, US 23rd Medical group (*n* = 12): physicians, physician assistants, nurse practitioners, physical therapists Active duty members (*n* = 4500)	“Physical Therapy First” orthopedic performance improvement initiative designed within Consolidated Framework for Implementation Research model Improve appropriate referrals and decrease inappropriate resource utilization for musculoskeletal injuries Initiative endorsed and facilitated by leadership to include the Medical Group Commander, the Chief of Staff and the full executive staff Engage professional staff (physicians, physician assistants, nurses, therapist) to develop consensus on proposed changes
Interventions targeted at healthcare workers	Educational materials	Cretin et al., 2001 [32], mixed methods	Army community hospitals (*n* = 3), Army medical center (*n* = 1), Great Plains Region, US Multidisciplinary implementation teams Teams (*n* = 4)	Integration of Department of Defense/Veteran Affairs low back pain clinical practice guideline (algorithms with annotations, discussion, references to graded evidence) Low back pain toolkit Patient education materials (brochures, curricula for classes, videos, CD-ROMs web sites) Physician education materials (annotated guideline, patient case examples, videos, CD-ROM, continuing medical education modules, Internet resources), documentation forms, drug formulary, recommended devices, or equipment Team Manual: develop and monitor a guideline implementation plan
		Feuerstein et al., 2006 [33], cross-sectional	Military Health Service healthcare services within the continental US, fiscal years 1998–2002 Military Health Service beneficiaries, age 18–65, who completed a Health Care Survey of Department of Defense	Implementation of clinical practice guideline for the diagnosis and management of acute low back pain System-wide educational efforts across facilities and providers Website: clinical practice guideline downloadable and printable, clinical practice guideline key elements, tools to facilitate implementation
	Educational meetings	Cretin et al., 2001 [32], mixed methods	Army community hospitals (*n* = 3), Army medical center (*n* = 1), Great Plains Region, US Multidisciplinary implementation teams Teams (*n* = 4)	Integration of Department of Defense/Veteran Affairs low back pain clinical practice guideline (algorithms with annotations, discussion, references to graded evidence) Teams attend 1 1/2 day workshop to review low back pain clinical practice guideline and toolkit Introduction of low back pain clinical practice guideline to primary care providers and other clinic staff
		James et al., 1981 [38], mixed methods	US army hospital, US Army Health Services Command data Physical therapists (*n* = 5) Active duty military (*n* = 3291)	Expanded physical therapist role as primary screener of musculoskeletal conditions Physical therapists performing musculoskeletal evaluations must complete 2-week musculoskeletal Assessment Course at US Army Academy of Health Sciences or civilian equivalent
		Larsson et al., 2012 [44], non-experimental	Swedish Armed Forces who started military training: 2003: *n* = 120 (artillery) 2004: *n* = 356 (ranger) 2004: *n* = 407 (engineer)	Musculoskeletal Screening Protocol Officers received training in ergonomics, recognizing musculoskeletal problems, first aid for musculoskeletal injuries, and exercise physiology through classroom instruction and practical exercises
		Lillie, 2010 [40], case report	Military treatment facility, US US Navy Petty Officer, age 40	Interdisciplinary management of low back pain Primary care manager visited, and medicine residents observed in chiropractic clinic Chiropractor provided in-service Chiropractor on sports medicine and research teams
	Continuous quality improvement	Cretin et al., 2001 [32], mixed methods	Army community hospitals (*n* = 3), Army medical center (*n* = 1), Great Plains Region, US Multidisciplinary implementation teams Teams (*n*, range 7–19)	Integration of Department of Defense/Veteran Affairs low back pain clinical practice guideline (algorithms with annotations, discussion, references to graded evidence) Develop action plan by site for introduction and implementation of low back pain clinical practice guideline Run small-scale test prior to implantation on a wide scale Utilize Plan-Do-Study-Act Cycles to refine change ideas and build support for facility wide adoption
		Feuerstein et al., 2006 [33], cross-sectional	Military Health Service healthcare services within the continental US, fiscal years 1998–2002 Military Health Service beneficiaries, age 18–65, who completed a Health Care Survey of Department of Defense Beneficiaries	Implementation of clinical practice guideline for the diagnosis and management of acute low back pain Manual for facility champions—7 step implementation process: (1) importance of knowing clinical practice guideline elements; (2) assess current practice; (3) compare current practice with clinical practice guideline recommendations; (4) identify gaps in current practice; (5) develop action plan to close gaps; (6) implement plan; (7) develop a system to monitor practice change
		Green et al., 2010 [36], case report	Naval hospital, US US Marine Corps F/A-18 aviation instructor, age 38	Interdisciplinary management of neck pain Close working relationship between flight surgeon and chiropractor ensures appropriate modalities, consistent follow-up, and adherence to regulations
		James et al., 1981 [38], mixed methods	US army hospital, US Army Health Services Command data Physical therapists (*n* = 5) Active duty military (*n* = 3291)	Expanded physical therapist role as primary screener for musculoskeletal conditions To assess overall quality of care provided by physical therapists: treatment records reviewed for legibility, completeness, medical appropriateness
	Communities of practice	Cretin et al., 2001 [32], mixed methods	Army community hospitals (*n* = 3), Army medical center (*n* = 1), Great Plains Region, US Multidisciplinary implementation teams Teams (*n*, range 7–19)	Integration of Department of Defense/Veteran Affairs low back pain clinical practice guideline (algorithms with annotations, discussion, references to graded evidence) Teams encouraged to share information about successes and failures through video conferences, teleconferences and e-mail list servers, to incorporate change recommendations to the centrally disseminated toolkit
	Local opinion leaders	Feuerstein et al., 2006 [33], cross-sectional	Military Health Service healthcare services within the continental US, fiscal years 1998–2002 Military Health Service beneficiaries, age 18–65, who completed a Health Care Survey of Department of Defense Beneficiaries	Implementation of clinical practice guideline for the diagnosis and management of acute low back pain Identification of clinical practice guideline advocate

*MD* medical doctor, *RCT* randomized control trial, *US* United States

**Table 2:** Barriers and facilitators of implementing musculoskeletal programs of care in active military using capability, opportunity, motivation-behavior (COM-B) system

	Facilitators	Barriers	Author(s), year
Capability			
Psychological capability (knowledge of psychological skills, strength or stamina to engage in the necessary mental processes)	DC treated service member with respect and concern DC able to respond to patient questions	Patient uncertainty regarding recovery expectations	Boudreau et al., 2006 [29]
		No formal training sessions for nurses, medics, physician assistants, and other support staff Uncertainty in applying CPG in multiple ailment cases	Cretin et al., 2001 [32]
	Chiefs of Professional Service, Department of Clinics and Radiology believed that the PT demonstrated capability to provide quality medical care in the screening role		James et al., 1975 [37]
	PTs with specialized training in musculoskeletal evaluation		James et al., 1981 [38]
	Use of current procedural terminology (CPT) code for patient education because reassurance and information demonstrated to be effective for spine conditions (this code not consistently used for spine cases) Use of specific coding by all members of Spine Team to differentiate care from that of other providers Cases that present with a premorbid psychological or psychiatric diagnosis should be identified because different outcomes may be expected		Ziemke et al., 2015 [42]
Physical capability (physical skill, strength, or stamina)			
Opportunity			
Physical opportunity (opportunity afforded by the environment involving time, resources, locations, cues, physical “affordance”)	Direct access to x-rays in hospital	Medical referral required for CT scan, MRI, or other diagnostic tests Improper equipment, e.g., medical treatment tables provided by the hospital rather than chiropractic tables	Boudreau et al., 2006 [29]
	Immediate on-site consultations between sport medicine physicians, athletic trainer, PT		Brawley et al., 2012 [30]
		Decreased patient privacy associated with open-bay configuration of the Sports and Medicine Reconditioning Team (SMART) clinic model	Brawley et al., 2012 [30]
		“Backs to Work” program modified from 5 to 3 days as patients unwilling to spend time away from work or unable to secure complete release from duty for treatment	Campello et al., 2012 [31]
		Different low back pain diagnostic codes made it difficult to compare across sites. Resolved by having sites agree to a single ICD-9 code Staff turnover resulted in repeated training Delays in distributing toolkit items Difficulty accessing web-based system to facilitate information exchange Differences in medical and administrative assets	Cretin et al., 2001 [32]
		Health providers available and ideally with primary care or first point of contact, e.g., PTs in separate department and inaccessible when needed (author)	Feuerstein et al., 2006 [33]
	DC in same clinic with PT		Green et al., 2006 [35]
		Limited equipment, e.g., no dual inclinometry for range of motion assessment	Green et al., 2010 [36]
		Too little time available for individual patients (increased workload without an increase in staffing) Lack of scheduling and resultant cyclic nature of workload Poor examination facilities Overall troop strengths, troop activities, weather conditions and epidemiological status of population influence number of visits to PT clinic	James et al., 1975 [37]
		Legibility problems with PT hand writing	James et al., 1981 [38]
	Development of MSK team and a training room created in the recruit medical clinic		Kelly et al., 1997 [39]
	Electronic medical record has built in referral process for specialty services	Electronic medical record maintained in a secure network and are unavailable to off-base providers; thus, applicable notes need to be delivered	Lillie, 2010 [40]
	Some care shifted to local private PT managed care network (to offset increased workload from “Physical Therapy First” approach) Sharing DoD resources through interagency collaboration PT as first line of care PT Director gatekeeper for all MSK consults requested by Primary Care staff Conservative treatment exhausted prior to referral to orthopedic specialty Allow specialty care referral to US Navy Jacksonville Orthopedic Department instead of private managed care network Active duty orthopedic consults consolidated daily by the Resource Management Officer Primary care provider informed patients that an orthopedic referral would occur after consultation with the MSK team		McGee et al., 2017 [43]
	Development of MSK Soldier Readiness Processing (SRP) Pathway to expedite access to MSK team (PT, physiatrist, sports medicine physician) among soldiers returning from deployment with MSI	Constant turnover of military personnel. Leaders are usually only in their position for 1–3 years, which means in a 5–7-year period you can have a complete turnover of staff. This leads to ongoing reinvention and makes it very difficult to gather traction for something that will last for a decent amount of time. Cannot assume that current leaders’ priorities and goals will be the same as the follow-on leader (author)	Rhon et al., 2017 [41]
	Develop a system for triaging service members with spine conditions to the Spine Team for care early after injury onset Use an evidence-based algorithm to allocate treatment DC part of primary care Spine Team (DC, PT, orthopedic surgeon or physiatrist) (author) DC is direct access while PT is not (author) DC saw most cases initially, would do a trial of therapy and then either discharge or refer to PT (author)	Delay in initiation of care for spine conditions, suggest that the condition was chronic before the Spine Team saw the patient Gaps in patterns of care: service members with spine conditions received follow-up conservative care from their operational medical team, which is not always reflected in the Composite Health Care System records DC saw one patient at a time, compared to PT who saw 2–3 patients at a time (author) Need a clear interdisciplinary team protocol, as well as an algorithm to avoid service duplication (author) Personnel turnover is a challenge for continuation of service implementation (author)	Ziemke et al., 2015 [42]
Social opportunity (opportunity afforded by interpersonal influences, social cues, and cultural norms that influence the way that we think about things, e.g., the words and concepts that make up our language)	Cognitive behavioural therapy included education about how psychosocial variables affect pain, relaxation training, modification of maladaptive beliefs, and problem solving		Campello et al., 2012 [31]
		Competing demands for resources and staff time Sites were slow to establish monitoring procedures, in part due to delays in providing “official” system-wide low back pain metrics	Cretin et al., 2001 [32]
	Advocate for low back pain CPG		Feuerstein et al., 2006 [33]
	Flight surgeon coordinated ordering and follow-up of clinical consults PT and DC communication to ensure non-duplication of service		Green et al., 2006 [35]
	Close working relationship between flight surgeon and DC	Suboptimal treatment frequency due to scheduling conflicts	Green et al., 2010 [36]
	Formal weekly meetings to discuss progress of more seriously injured recruits		Kelly et al., 1997 [39]
	DC attend weekly meeting with specialty providers to discuss specific cases		Lillie, 2010 [40]
	Specialists exchange evidence-based approaches to care Primary care manager visited DC clinic and was familiar with the approach to care Family medicine residents’ observations in DC clinic DC provide in-service presentations		
	Endorsed and facilitated by leadership to include the Medical Group Commander, the Chief of Staff, and the full executive staff Implementation champion (PT) Professional staff (physicians, physician assistants, nurses, and therapists) engaged in forum to develop consensus on proposed protocol changes Professional staff briefed with background and supporting evidence at monthly staff meeting to promote buy-in Clinical interventions and pathways reviewed each quarter Professional staff received feedback on clinical metrics and issues as they arose Audit and feedback reporting to professional staff to reinforce that their referral behaviors were being monitored Clinical autonomy of primary care teams respected Emphasizing benefits for each stakeholder group: improved surgical/procedural throughput for network and military orthopedic specialists; transparency and constant reporting enabled primary care staff to observe benefits associated with following evidence-based guidelines	Fear that changes would result in increased burden to the provider, offset by single step to minimize workflow disruption and protected PT time for chart review	McGee et al., 2017 [43]
Motivation			
Reflective (reflective process involving plans (self-conscious intentions) and evaluations (beliefs about what is good and bad))	Buy-in from authorities as well as clinicians affected by the program (author)		Campello et al., 2012 [31]
	Teams moderately motivated to implement CPG due to resistance to the concept of guidelines, uncertainty about the implementation demonstration, and concerns about increased workload	Previous experience with guidelines Expected rewards from implementation	Cretin et al., 2001 [32]
		Low rate of adherence to low back pain CPG likely resulted from providers assuming that most cases of low back pain resolve spontaneously Primary care providers thought they knew how to manage low back pain (author) Primary care providers did not think the low back pain CPG was defensible despite being evidence-based (author) Providers heavily influenced by patient desires, e.g., patient requests MRI even though CPG was clear that MRI was not indicated (author)	Feuerstein et al., 2006 [33]
		Coping with too-often-obvious gain phenomena in many patients, e.g., obtain benefits or be excused from duty	James et al., 1975 [37]
	Create a plan, then brief it at varying levels until you reach authorities who can make it happen. Much of it is salesmanship, doing your homework to answer the “business” questions, make sure it addresses “perceived needs,” etc. (author)	Outcomes are not captured very well in military health system. Varying opinions as to what constitutes “value” and what should be measured. A system to create outcome measures needs to be created, but the direct cost/benefit is uncertain therefore difficult to sell. Assessed patient satisfaction (which is not good measure of quality), costs, access to care, and leakage to civilian settings (goal to keep as many patients in the military system and maintain access times, so not referred to civilian settings) (author)	Rhon et al., 2017 [41]
	Primary care and PT teams worked collaboratively in pre-existing culture of trust and mutual sharing		McGee et al., 2017 [43]
Automatic (automatic processes involving emotional reactions, desires (wants and needs), impulses, inhibitions, drive states, and reflex responses)		Most flight surgeons (designated first point of contact) are accustomed to collaborating with physiatrists and PTs but not DCs	Green et al., 2006 [35]

Refer to Table 1 for the study design, clinical setting, and participant information

*BTW* backs to work, *CPG* clinical practice guideline, *CT* computed tomography, *DC* chiropractor, *DoD* Department of Defense, *LIMDU* limited duty, *MRI* magnetic resonance imaging, *MSI* musculoskeletal injuries, *MSK* musculoskeletal, *PT* physical therapist

**Table 3:** Implementation outcomes of musculoskeletal programs of care in active military using the implementation research outcomes taxonomy by Proctor et al. [23]

		Author(s), year
Implementation outcomes		
Acceptability	Physicians tested the preprinted documentation form and concluded that the form was easy to use and shortened the time to process patients. Subsequently, primary care physicians readily accepted the use of the new form	Cretin et al., 2001 [32]
	Concept and quality of care acceptable to patient, physician, and PT Patients preferred direct referral to PT	James et al., 1975 [37]
	The musculoskeletal team has successfully created an environment of systematic collaboration	Kelly et al., 1997 [39]
	No reported adverse events with the “Physical Therapy First” approach	McGee et al., 2017 [43]
Appropriateness	Allows early and accurate diagnosis Allows early and aggressive reconditioning Coordinated care between providers Bridges the gap between primary care and orthopedic surgeons	Brawley et al., 2012 [30]
	Recruits referred earlier in the course of their injuries	Kelly et al., 1997 [39]
	Appropriate referrals: > 55% actual surgical cases referred to orthopedic surgeons (vs. 10–15% prior to implementing the “Physical Therapy First” approach)	McGee et al., 2017 [43]
Costs	LIMDU boards that resulted in PEBs decreased following implementation of the SMART clinic model Significant increases in the number of patient encounters at the sports medicine clinics Decrease in referrals to orthopedic surgeon	Brawley et al., 2012 [30]
	Decreased number of PT sessions required for rehabilitation	Kelly et al., 1997 [39]
	Savings over 6 months $162.6K USD attributed to proper utilization of the “Physical Therapy First” approach	McGee et al., 2017 [43]
Feasibility	Given appropriate staffing levels and adequate space, all PTs and other providers believed the program should be adopted	James et al., 1975 [37]
Fidelity	Not applicable	
Penetration	Expanded PT LBP MSK evaluation role gained wide acceptance within the Army Medical Department PTs now provide primary evaluations for the whole spectrum of MSK problems	James et al., 1981 [38]
Sustainability	“Backs to Work” program with a modified schedule continues at Naval Medical Center, Portsmouth	Campello et al., 2012 [31]
	Continued analysis of LBP CPG implementation	Cretin et al., 2001 [32]
	MSK screening protocol continued as planned in 1 unit 1 year later	Larsson et al., 2012 [44]
Service outcomes		
Efficiency	Increased number of patient encounters; decreased referrals to orthopedic surgery clinic; decreased percentage of patients recommended for physical evaluation boards from limited duty periods	Brawley et al., 2012 [30]
	Utilization patterns during 6-week follow-up after CPG implementation: decreased referrals to PT/DC; no effect on specialty referrals	Cretin et al., 2001 [32]
	CPG adherence was associated with lower health costs	Feuerstein et al., 2006 [33]
	Total outpatient visits, number of back patient visits, time expended by PT in attending LBP patients, identification of disease and patient categories for evaluation, orthopedist appraisal	James et al., 1975 [37]
	Less than 4% of active duty patients with MSK complaints first evaluated by the PT subsequently required orthopedic consultations	James et al., 1981 [38]
	Economical way to treat significant numbers of injured recruits (reduced number of PT sessions required to return an injured recruit to training, decreased total lost time for injuries requiring PT). Saved the Navy millions of dollars in recovered lost training time and retained, return to full training; number of PT sessions needed; recruit attrition; lost duty days of training.	Kelly et al., 1997 [39]
Safety		
Effectiveness	Duty status	Campello et al., 2012 [31]
	Return to duty	Green et al., 2010 [36]
	Resumed normal work activities, released from care	Lillie et al., 2010 [40]
	Disability (proportion of active-duty service members seeking treatment for a work-disabling spine condition that results in the assignment of a first-career limited-duty status decreased), attrition (proportion of individuals assigned a first-career limited-duty status for a work-disabling spine condition who were referred to a Physical Evaluation Board (no observed effect))	Ziemke et al., 2015 [42]
Equity		
Patient-centeredness		
Timeliness	Sports Medicine and Reconditioning Team SMART clinic improved MSK care access	Brawley et al., 2012 [30]
	Form shortened the time to process patients Timelines of toolkit production improved over time	Cretin et al., 2001 [32]
	Decreased wait times for LBP patients	James et al., 1975 [37]
	Duration of evaluation twice as long as non-evaluation PT visits Substantial physician hours saved	James et al., 1981 [38]
Client/patient outcomes		
Symptomology	Pain, psychological distress at 12 weeks, function, fitness	Campello et al., 2012 [31]
	CPG adherence was associated with improved perceived general health (HCSDB)	Feuerstein et al., 2006 [33]
	Back-related pain (NRS), global improvement	Goertz et al., 2013 [34]
	Pain (VAS)	Green et al., 2006 [35]
	Pain-free (NRS) at 8 weeks	Green et al., 2010 [36]
	Subjective complaints resolved	Lillie et al., 2010 [40]
Function	Participants reported lower disability and pain. All (in both arms) returned to duty at 12 weeks	Campello et al., 2012 [31]
	CPG adherence was associated with functional outcome: released with/without duty limitations, lower levels of disability	Feuerstein et al., 2006 [33]
	Physical functioning (RMDQ)	Goertz et al., 2013 [34]
	Disability (RMDQ)	Green et al., 2006 [35]
	No disability (NDI) at 8 weeks	Green et al., 2010 [36]
	Reduced premature discharge from training	Larsson et al., 2012 [44]
Satisfaction	Satisfaction: 94.2% satisfied with chiropractic care; none dissatisfied Factors associated with lower satisfaction with chiropractic care: older age, presenting complaint of knee pain Referring Physician Feedback Survey: 80.0% satisfied with chiropractic services	Boudreau et al., 2006 [29]
	CPG adherence was associated with higher levels of patient satisfaction	Feuerstein et al., 2006 [33]
	Higher patient satisfaction in CMT + SMC (mean 8.9/10 vs. 5.4/10 in SMC alone)	Goertz et al., 2013 [34]
	Job satisfaction, PTs’ self-appraisal of competence, difficulties, professional adequacy; patient satisfaction	James et al.,1975 [37]
	PTs preferred: expanded role; MSK patients interspersed within overall practice	James et al., 1981 [38]
	Patient satisfaction was very high (*n* = 179), median score 5/5 (completely agree or completely satisfied)	Rhon et al., 2017 [41]

Refer to Table 1 for the study design, clinical setting, and participant information

*BCT* brigade combat teams, *CPG* clinical practice guideline, *CSH* combat support hospital, *HCSDB* Health Care Survey of DOD Beneficiaries, *CMT* chiropractic manipulative therapy, *LIMDU* limited duty, *MST* musculoskeletal team, *NDI* neck disability index, *NRS* numerical rating scale, *PEBs* physical evaluation boards, *PT* physical therapist, *RMDQ* Roland-Morris Disability Questionnaire, *SMC* standard medical care, *VAS* visual analogue scale

### Implementation interventions for musculoskeletal programs of care

The studies lacked explicit descriptions of the process of implementation of interventions for musculoskeletal care. Nonetheless, we used the information available to classify the interventions according to EPOC [21] as delivery arrangements [29–31, 34–44] and implementation strategies [32, 33, 36, 38, 40, 43, 44] (Table 1).

Health care was commonly delivered in a coordinated and multidisciplinary fashion, facilitated by specific referral systems, care pathways, roles and responsibilities, communication methods, use of technology (e.g., electronic health records), and infrastructure (e.g., the co-location of team members). We observed three distinct methods of delivering multidisciplinary care, each with different gatekeepers. A common approach was that a medical physician or specialist was required to make referrals to other musculoskeletal healthcare providers such as chiropractors, physical therapists, and psychologists [29, 35, 36, 40–42]. In contrast, other studies used non-physicians as gatekeepers such as the “Physical Therapy First” [43] approach [31, 37, 38]. Finally, other studies described teams collectively assessing and managing military members with musculoskeletal disorders [30, 39].

Strategies to implement interventions for managing musculoskeletal disorders included those that targeted healthcare organizations [33, 43] or healthcare workers [32, 33, 36, 38, 40]. For example, implementation of interventions may be facilitated by having strong support from the organizational leadership. Strategies aimed at healthcare workers may facilitate the implementation of clinical practice guidelines, for example, such as providing workers with educational materials and meetings and identifying local opinion leaders who can advocate for the use of guidelines. One study highlighted that implementation is an iterative process. For example, the implementation of a low back pain clinical practice guidelines in four military medical settings used successive Plan-Do-Study-Act (PDSA) cycles and encouraged teams to continually evaluate successes and failures. This evaluation allowed adjustments and retesting before full implementation on a broad scale [32]. To target healthcare organizations, another study developed and assessed a process to implement low back pain clinical practice guidelines in the military healthcare system. Leadership support was established, as well as a handbook to facilitate guideline adoption within the system [33].

### Barriers and facilitators of implementation

#### Capability

The main barriers in this domain were psychological not physical [29, 32, 37, 38, 42]. For example, barriers to guideline implementation for low back pain included healthcare clinicians not fully understanding how to apply the guideline for patients with multiple conditions. Similarly, few formal training opportunities were available to nurses, physician assistants, and other support staff [32]. Facilitators to implementation included consistent coding for diagnoses and procedures across healthcare providers and sites to facilitate common language [32, 42], knowledge of conditions that may delay recovery from spinal pain [42], and advanced training and expertise regarding the management of musculoskeletal conditions [38].

#### Opportunity

A number of studies demonstrated that implementation was affected by opportunity [29–33, 35–37, 39–43], and often, the physical environment impacted the social environment. For example, the integration of chiropractic services in military treatment facilities was facilitated by having chiropractors located in the same clinic as other providers. This enabled continual communication with other providers to ensure appropriate treatment, non-duplication of services, consistent follow-up, and that regulations were closely followed [35, 36]. Other studies also demonstrated that physical proximity to different providers allowed for immediate on-site consultations, as did regular meetings to discuss cases and evidence-based approaches to care [30, 39–42]. Implementation was facilitated by sharing resources where possible [43] and having direct access to physical therapists [43]. Implementation was also facilitated by identifying an advocate or champion for the program, which can be considered to be a social opportunity [33].

In some studies, factors in the physical environment hindered implementation, such as having access to limited or improper facilities, equipment, or systems [29–32, 36, 37]. Having limited staff impacts implementation [32, 37]. High staff turnover, for instance, requires repeated training for the intervention [32]. Implementing an interdisciplinary program of care can be difficult without the appropriate systems in place to facilitate information exchange [32, 40, 42]. Lillie et al. described how military electronic medical records are maintained in a secure network [40]. However, if a service member was referred off-base for care, that provider did not have direct access to the electronic health care notes, and arrangements had to be made for the applicable notes to be delivered and scanned into the external provider record. Finally, gaps in patterns of care can pose a challenge for implementation. A delay in the initiation of care for service members with spine conditions resulted in spine conditions that were already chronic before the interdisciplinary spine team could assess the patient [42].

#### Motivation

Motivational barriers can hinder implementation. An example of a motivational barrier was described by Green et al. [35]. Most flight surgeons, who are typically designated as the first points of contact for military pilots, are accustomed to collaborating with physical therapists and physiatrists rather than with chiropractors, thus challenging interdisciplinary collaboration. Reflective motivational barriers were illustrated in three studies. From survey data, teams were only moderately motivated to implement guidelines because they resisted the guidelines concept; were uncertain about the guideline implementation demonstration; were concerned about increased workload; were influenced by previous negative experiences with practice guidelines; and expected rewards from implementation such as recognition from the leadership [32]. Clinicians might have been reluctant to provide the variety of services recommended in the low back pain clinical practice guideline because they were influenced by clinical experiences and by assumptions that most cases of low back pain resolve spontaneously [33]. Healthcare providers working collaboratively in a pre-existing culture of trust and mutual sharing is an example of a reflective motivational facilitator [43].

### Outcomes of implementation interventions

Service and patient outcomes were more frequently reported compared to implementation outcomes in the included studies. The service outcomes reported included timely access to care [30, 37, 38], efficiency with respect to patient encounters and specialist referrals [30, 32, 33, 37–39, 42], and effectiveness of care (e.g., duty status, and discharge from care) [30, 31, 36, 39, 40]. With respect to patient outcomes, investigators reported improved symptomatology such as pain and perceived general health [31, 33–36, 40], function including disability and physical fitness [31, 33–36, 38], and patient and provider satisfaction [29, 33, 34, 37, 41].

Four studies provided evidence that the implemented programs were acceptable and appropriate to healthcare providers or patients [30, 32, 37, 43]. Implementing interdisciplinary management of musculoskeletal injuries in a training room open-bay approach allowed for early and accurate diagnosis, early and aggressive reconditioning, coordinated care between providers, and bridging of the gap between primary care and orthopedic surgeons [30]. The concept and quality of care from having a physical therapist in an expanded primary care role was acceptable to the physician and physical therapist, and patients preferred direct access to the physical therapist clinic [37]. The feasibility of this program and others like it, and its adoption, penetration, and sustainability, was demonstrated [38]. Physical therapists now provide primary evaluations over the entire spectrum of musculoskeletal problems in US military health settings. Sustainability was demonstrated with programs such as the “Backs to Work” program in the US [31] and the “Musculoskeletal Screening Protocol” in Sweden [44]. Costs savings were also realized with a “Physical Therapy First” approach [43].

### Consultation

We emailed all corresponding authors of the studies included in our review and were able to connect with four authors who shared their insights regarding interdisciplinary teams managing musculoskeletal disorders in the active military. They emphasized the importance of having clear protocols, such that all team members know exactly when and how to intervene. To do this, they stated that care pathways need to be developed that do not allow for the duplication of services, and demonstrate cost-effectiveness of care. One author discussed the importance of relationship building among team members. While this may take some time, it can be easily achieved with agreed upon team protocols and care pathways. One author also pointed out that having advocates for a particular program of care is not enough if the environment is not conducive to the program. For instance, providers should be physically situated together in a team and available at the first point of patient contact. In his experience, physical therapists were often in their own departments and thus, inaccessible when needed.

Authors also discussed that one of the largest barriers to program implementation is the constant turnover of military personnel, making it difficult to implement let alone sustain programs. One author suggested the importance of having an interdisciplinary team of civilian providers that is stationary and has the opportunity to build good working relationships. These civilian providers would serve as “pillars” needed to sustain a musculoskeletal program of care.

Finally, one author spoke about the need to invest in an infrastructure that collects important outcome measures from military patients. In his experience, important outcomes are not well captured within the military health system. There are varying opinions as to what constitutes “value” and therefore what should be measured. Some outcomes considered are patient satisfaction, costs, access to care, and having to out-source to civilian settings. He discussed that military providers want to keep patients in the military health system; however, if access times become too long, patients are referred to civilian providers.

We consulted organizations (Canadian Armed Forces and the Canadian and Ontario Chiropractic Associations) and other experts during a summer institute (Knowledge Translation Canada, June 2017) for their perspectives and experiences regarding barriers and facilitators to health program implementation. Stakeholders and experts suggested that barriers included lack of motivation and knowledge on how to change current practice patterns and behaviors, and time constraints of implementing changes, while facilitators included incentives (e.g., monetary, professional) and audit and feedback processes.

## Discussion

To our knowledge, this is the first scoping study to identify implementation interventions for musculoskeletal programs of care in the active military and barriers, facilitators, and outcomes of implementation.

The most common interventions implemented in the military were delivered by coordinated multidisciplinary teams with good communication practices. Indeed, multidisciplinary interventions have increased over the last few decades given that musculoskeletal disorders and disability are influenced by somatic pathology, and psychological and social factors [45]. A common implementation strategy was using educational materials. There is little comparative effectiveness research upon which to base the selection of dissemination strategies [46]. However, there is some evidence to support a multi-component approach such as use of educational meetings/materials and opinion leaders [47, 48].

Of the studies reporting barriers and facilitators to implementation, most mapped onto the opportunity component of the COM-B model, which refers to environmental factors (physical or social) facilitating or impeding program implementation. Our findings are consistent with previous studies assessing barriers to program implementation using the COM-B model, with barriers also mapping onto the opportunity component. These include studies aiming to design implementation interventions to improve smoking cessation care for pregnant indigenous smokers [49], to improve hearing-aid use in adult auditory rehabilitation [50], to reduce imaging for low back pain [51], and others [52, 53]. Identifying barriers and facilitators to program implementation using a system such as COM-B, and subsequently implementing corresponding behavior change techniques, may help to improve the clinical effectiveness and impact of programs of care [54, 55]. Our findings also indicate that some programs of care for musculoskeletal disorders in the active military were acceptable, appropriate, feasible, and sustainable.

Our study has research implications. Future research should better *describe* the implementation steps of programs of care. Some studies in our review provided little information describing *how* programs of care were implemented. Implementation strategies are complex social interventions addressing multifaceted processes within interpersonal, organizational, and community contexts [56–58]. Therefore, as with clinical intervention research, implementation interventions need to be precisely described to enable measurement and reproducibility [59] of their components [60]. Proctor and colleagues proposed guidelines for naming, defining, and operationalizing implementation interventions in terms of seven dimensions: actor, the action, action targets, temporality, dose, implementation outcomes addressed, and theoretical justification [60]. Following these guidelines may improve the understanding of how, why, when, and where implementation interventions work. Having well-described implementation interventions should allow researchers to study their effectiveness on implementation in properly conducted trials; this is a major research priority.

While a multidisciplinary approach was common, further research should explore the optimal components of this approach. For example, which types of healthcare providers/community workers should be part of the team, do they need to be co-located, and what should their roles be to optimize efficiency, safety, and clinical- and cost-effectiveness of musculoskeletal care? For instance, evidence suggests that extended scope physiotherapists provide equal or better care than physicians for musculoskeletal conditions in terms of diagnostic accuracy, treatment effectiveness, use of healthcare resources, economic costs, and patient satisfaction [61, 62]. Interprofessional musculoskeletal models of care and the extension of the scope of practice for allied health professionals (e.g., therapist-led care) are becoming priorities in high-income countries due to rising healthcare costs, physician shortages, aging of the population, and the increased prevalence of chronic diseases [63]. In our current review, we observed that some team members were co-located and this was useful for consultation. Further research might explore whether team members that manage musculoskeletal conditions should be co-located or if they function similar to remote/virtual teams in terms of safety, efficiency, and effectiveness. Nonetheless, “optimal effectiveness of clinical care teams requires a culture of trust; shared goals; effective communication; and mutual respect for the distinct skills, contributions, and roles of each member” [64].

Most of the barriers and facilitators of implementation mapped onto the opportunity component of the COM-B model; thus, they are generally modifiable. Future research should investigate the effectiveness of behavior change techniques that target these barriers [22, 65]. For example, barriers to clinical practice guideline compliance, some of which were identified in the studies we reviewed, include awareness, familiarity with the content, skills, difficulty in changing usual practice, equipment, space, time, staff, and financial resources [66]. Electronic health records that support integration of guidelines at the point of care, clinical decision support tools, and financial incentives/compensation are some ways to alter the physical environment to promote guideline compliance [67, 68]. In addition, leadership support and opinion leaders can create a social environment that facilitates guideline uptake by addressing provider beliefs and attitudes [69].

Finally, regarding outcomes, evaluating and reporting implementation outcomes should precede the evaluation and report of service and patient outcomes [23]. A number of implementation outcomes were either not evaluated or had limited evaluation such as fidelity, penetration, sustainability, adoption, and costs. Further, valid and reliable measures of implementation outcomes are required and should be used consistently by researchers; work is underway in this area [70].

### Strengths and limitations

Our scoping review has strengths. Our health sciences librarian conducted a broad and methodologically rigorous literature search, which was reviewed by a second librarian. We outlined detailed inclusion/exclusion criteria to identify relevant studies, pairs of independent trained reviewers screened the literature, and we used theoretical frameworks (EPOC, COM-B, Proctor et al.’s taxonomy of implementation research outcomes) to map and synthesize our findings. Potential limitations include the potential for missed studies not identified through the search strategy, and the use of studies published in English only. However, evidence suggests that this language restriction would not have significantly altered our results [71–73]. Another limitation was that we did not qualitatively analyze comments from authors of studies, which may have provided deeper insight into our scoping review results.

## Conclusion

Musculoskeletal disorders are a leading cause of disability in the military and effective treatment strategies are required to improve return to duty and maintain operational readiness. However, implementing programs of care is challenging given the complexity of the military healthcare system. We synthesized the available scientific literature regarding implementation interventions for musculoskeletal programs of care in the active military, and the barriers, facilitators, and outcomes of implementation. Further research is needed to better understand the various components and players of implementation interventions, how to overcome barriers to implementation, effectiveness of implementation interventions, and on implementation outcomes and their measurement. Once a program of care is successfully implemented, the ultimate goal is to determine whether it improves important patient outcomes such as recovery and return to duty.

## Data Availability

Not applicable

## Appendix

### Search strategies

MEDLINE Search Strategy

Search run April 25, 2017 in Ovid MEDLINE: Epub Ahead of Print, In-Process & Other Non-Indexed Citations, Ovid MEDLINE® Daily and Ovid MEDLINE® 1946-Present; results (English): 1276

Search run June 1, 2018 in Ovid MEDLINE(R) In-Process & Other Non-Indexed Citations, Ovid MEDLINE(R) Daily and Ovid MEDLINE(R) 1946 to Present; 137 results

1. Military Personnel/

2. United States Department of Defense/

3. United States Department of Veterans Affairs/

4. Warfare/

5. active duty.ab,ti.

6. air force*.ab,ti.

7. armed forces.ab,ti.

8. (army or armies).ab,ti.

9. coast guard.ab,ti.

10. conscript*.ab,ti.

11. ((defence or defense) adj3 (department* or force*)).ab,ti.

12. limited-duty assignment*.ab,ti.

13. marching.ab,ti.

14. marine corps.ab,ti.

15. marines.ab,ti.

16. (military adj3 (base* or facilit* or installation* or personnel or population* or service*)).ab,ti.

17. ((navy or navies or naval) adj3 (base* or facilit* or installation* or personnel or population* or service*)).ab,ti.

18. sailor*.ab,ti.

19. soldier*.ab,ti.

20. submariner*.ab,ti.

21. "Department of Defense".ab,ti.

22. "Department of Veterans Affairs".ab,ti.

23. or/1-22 [**military terms]

24. Whiplash Injuries/

25. Neck Injuries/

26. Neck Pain/

27. Neck Muscles/in [Injuries]

28. exp Cervical Vertebrae/in [Injuries]

29. Radiculopathy/

30. exp Brachial Plexus Neuropathies/

31. exp Torticollis/

32. whiplash.ab,ti.

33. "neck injur*".ab,ti.

34. "neck pain*".ab,ti.

35. "neck ache*".ab,ti.

36. "neckache*".ab,ti.

37. "brachial plexus neuropath*".ab,ti.

38. torticollis.ab,ti.

39. or/24-38

40. exp Back Injuries/

41. exp Back Pain/

42. Coccyx/in [Injuries]

43. Intervertebral Disc Degeneration/

44. Intervertebral Disc Displacement/

45. Lumbar Vertebrae/in [Injuries]

46. Lumbosacral Region/in [Injuries]

47. Osteoarthritis, Spine/

48. Piriformis Muscle Syndrome/

49. Sciatica/

50. Spinal Diseases/

51. Spinal Stenosis/

52. (back adj3 (ache* or injur* or pain*)).ab,ti.

53. (backache* adj3 (injur* or pain*)).ab,ti.

54. (back pain or back-pain).ab,ti.

55. (lumbar disc* adj3 (extruded or degenerat* or herniat* or prolapse* or sequestered or slipped)).ab,ti.

56. (lumbar disk* adj3 (extruded or degenerat* or herniat* or prolapse* or sequestered or slipped)).ab,ti.

57. "low* back pain".ab,ti.

58. (lumbar adj3 (pain or facet or nerve root* or osteoarthritis or radicul* or spinal stenosis or spondylo* or zygapophys*)).ab,ti.

59. "Piriformis syndrome*".ab,ti.

60. (sacral adj2 pain*).ab,ti.

61. ((spine or spinal) adj4 (condition* or disable* or disabilit* or disorder* or pain or stenos?s)).ab,ti.

62. spondylosis.ab,ti.

63. or/40-62

64. Shoulder Pain/

65. exp Cumulative Trauma Disorders/

66. exp Median Neuropathy/

67. Shoulder Impingement Syndrome/

68. Shoulder Joint/in [Injuries]

69. Shoulder/in [Injuries]

70. exp Arm Injuries/

71. exp Hand Injuries/

72. Wrist Injuries/

73. Finger Injuries/

74. exp Tendinopathy/

75. Radial Neuropathy/

76. exp Ulnar Neuropathies/

77. Bursitis/

78. Thoracic Outlet Syndrome/

79. carpal tunnel syndrome.ab,ti.

80. (medial and (epicondylitis or epicondylosis or epicondylopathy)).ab,ti.

81. (lateral and (epicondylitis or epicondylosis or epicondylopathy)).ab,ti.

82. (shoulder* and (pain* or sprain* or strain* or injur* or impair* or impingement)).ab,ti.

83. (shoulder* and (tendinopathy or tendinitis or tendonitis or capsulitis)).ab,ti.

84. ((glenohumeral or scapul* or acromioclavicular) and (pain* or sprain* or strain* or injur*)).ab,ti.

85. (rotator cuff and (sprain* or strain* or tear* or bursitis tendinitis or impingement)).ab,ti.

86. ((supraspinatus or infraspinatus or subscapularis or teres minor or teres major or trapezius or deltoid or bicep* or bicipital or coracobrachialis) and (impingement or strain* or tear* or pain*)).ab,ti.

87. biceps tend?nitis.ab,ti.

88. painful arc.ab,ti.

89. frozen shoulder.ab,ti.

90. (shoulder and capsul* and (sprain* or tear*)).ab,ti.

91. (forearm* and (pain* or sprain* or strain* or injur* or impair*)).ab,ti.

92. (arm* and (pain* or sprain* or strain* or injur* or impair*)).ab,ti.

93. (wrist* and (pain* or sprain* or strain* or injur* or impair*)).ab,ti.

94. (hand* and (pain* or sprain* or strain* or injur* or impair*)).ab,ti.

95. (elbow* and (pain* or sprain* or strain* or injur* or impair*)).ab,ti.

96. "thoracic outlet syndrome*".ab,ti.

97. tennis elbow.ab,ti.

98. (rotator cuff and (injur* or disorder*)).ab,ti.

99. (median adj neuropath*).ab,ti.

100. (radial adj neuropath*).ab,ti.

101. bursitis.ab,ti.

102. "upper extremit* injur*".ab,ti.

103. ((radial or ulnar) adj neuropath*).ab,ti.

104. "cumulative trauma disorder*".ab,ti.

105. (repetit* and (strain* or sprain* or injur* or disorder*)).ab,ti.

106. or/64-105

107. exp Hip Injuries/

108. exp Leg Injuries/

109. exp Knee Injuries/

110. exp Foot Injuries/

111. exp Toes/in [Injuries]

112. Ankle Injuries/

113. Lateral Ligament, Ankle/in [Injuries]

114. Fasciitis, Plantar/

115. (lower and (extremit* or limb* or injur*)).ab,ti.

116. (ankle* and (sprain* or strain* or injur*)).ab,ti.

117. ((talofibular or calcaneofibular or calcaneotibial or tibio*) and (sprain* or strain* or injur*)).ab,ti.

118. (buttock* and (injur* or pain*)).ab,ti.

119. (foot and (injur* or pain*)).ab,ti.

120. (hip* and (injur* or pain*)).ab,ti.

121. (knee* and (injur* or pain*)).ab,ti.

122. (leg* and (injur* or pain*)).ab,ti.

123. (thigh* and (injur* or pain*)).ab,ti.

124. (toe* and (injur* or pain* or turf)).ab,ti.

125. "patellofemoral pain syndrome*".ab,ti.

126. tendinosis.ab,ti.

127. tendinopathy.ab,ti.

128. plantar fasciitis.ab,ti.

129. or/107-128

130. Musculoskeletal Diseases/

131. ((musculoskeletal or musculo-skeletal or MSK) adj4 (care or condition* or disabilit* or disorder* or injur* or pain or problem* or trouble*)).ab,ti.

132. 130 or 131

133. 39 or 63 or 106 or 129 or 132

134. Delivery of Health Care/

135. Delivery of Health Care, Integrated/

136. Health Planning/

137. Health Promotion/

138. Health Services Administration/

139. Hospitals, Military/

140. Integrative Medicine/

141. Interprofessional Relations/

142. Military Medicine/

143. Patient Care Management/

144. (approach* adj3 (collaborative or complementary or comprehensive or innovative or integrated)).ab,ti.

145. barrier*.ab,ti.

146. facilitator*.ab,ti.

147. ((health care or healthcare or health-care) adj3 (clinic or clinics or delivery or implement* or intervention* or military or model* or plan* or process* or program*or services or strateg* or system* or team*)).ab,ti.

148. (implement* adj3 (intervention* or model* or plan* or process* or program*or strateg* or system*)).ab,ti.

149. (innovative adj3 (intervention* or model* or plan* or process* or program*or strateg* or system*)).ab,ti.

150. (military adj3 (care or clinic or clinics or hospital* or medical or medicine or program*)).ab,ti.

151. (model* adj care).ab,ti.

152. ((integrated or interdisciplinary or interprofessional or multidisciplinary or multi-disciplinary) adj3 (care or clinic or clinics or implement* or intervention* or military or model* or plan* or process* or program*or strateg* or system*)).ab,ti.

153. (pathway* adj3 (clinical or care)).ab,ti.

154. primary health care.ab,ti.

155. (program* adj3 (assess* or evaluat*)).ab,ti.

156. or/134-155

157. 23 and 133 and 156

158. limit 157 to english language

### Cochrane Central Register of Controlled Trials Search Strategy

Search run April 26, 2017 in EBM Reviews - Cochrane Central Register of Controlled Trials March 2017; 56 results

Search run June 1, 2018 in EBM Reviews - Cochrane Central Register of Controlled Trials April 2018; 10 results

**Table:**

# ▼	Searches
183	limit 182 to english language
182	23 and 158 and 181
181	or/159-180
180	(program* adj3 (assess* or evaluat*)).ab,ti.
179	primary health care.ab,ti.
178	(pathway* adj3 (clinical or care)).ab,ti.
177	((integrated or interdisciplinary or interprofessional or multidisciplinary or multi-disciplinary) adj3 (care or clinic or clinics or implement* or intervention* or military or model* or plan* or process* or program*or strateg* or system*)).ab,ti.
176	(model* adj care).ab,ti.
175	(military adj3 (care or clinic or clinics or hospital* or medical or medicine or program*)).ab,ti.
174	(innovative adj3 (intervention* or model* or plan* or process* or program*or strateg* or system*)).ab,ti.
173	(implement* adj3 (intervention* or model* or plan* or process* or program*or strateg* or system*)).ab,ti.
172	((health care or healthcare or health-care) adj3 (clinic or clinics or delivery or implement* or intervention* or military or model* or plan* or process* or program*or services or strateg* or system* or team*)).ab,ti.
171	facilitator*.ab,ti.
170	barrier*.ab,ti.
169	(approach* adj3 (collaborative or complementary or comprehensive or innovative or integrated)).ab,ti.
168	Patient Care Management/
167	Military Medicine/
166	Interprofessional Relations/
165	Integrative Medicine/
164	Hospitals, Military/
163	Health Services Administration/
162	Health Promotion/
161	Health Planning/
160	Delivery of Health Care, Integrated/
159	Delivery of Health Care/
158	43 or 88 or 131 or 154 or 157
157	155 or 156
156	((musculoskeletal or musculo-skeletal or MSK) adj4 (care or condition* or disabilit* or disorder* or injur* or pain or problem* or trouble*)).ab,ti.
155	Musculoskeletal Diseases/
154	or/132-153
153	plantar fasciitis.ab,ti.
152	tendinopathy.ab,ti.
151	tendinosis.ab,ti.
150	"patellofemoral pain syndrome*".ab,ti.
149	(toe* and (injur* or pain* or turf)).ab,ti.
148	(thigh* and (injur* or pain*)).ab,ti.
147	(leg* and (injur* or pain*)).ab,ti.
146	(knee* and (injur* or pain*)).ab,ti.
145	(hip* and (injur* or pain*)).ab,ti.
144	(foot and (injur* or pain*)).ab,ti.
143	(buttock* and (injur* or pain*)).ab,ti.
142	((talofibular or calcaneofibular or calcaneotibial or tibio*) and (sprain* or strain* or injur*)).ab,ti.
141	(ankle* and (sprain* or strain* or injur*)).ab,ti.
140	(lower and (extremit* or limb* or injur*)).ab,ti.
139	Fasciitis, Plantar/
138	Lateral Ligament, Ankle/in [Injuries]
137	Ankle Injuries/
136	exp Toes/in [Injuries]
135	exp Foot Injuries/
134	exp Knee Injuries/
133	exp Leg Injuries/
132	exp Hip Injuries/
131	or/89-130
130	(repetit* and (strain* or sprain* or injur* or disorder*)).ab,ti.
129	"cumulative trauma disorder*".ab,ti.
128	((radial or ulnar) adj neuropath*).ab,ti.
127	"upper extremit* injur*".ab,ti.
126	bursitis.ab,ti.
125	(radial adj neuropath*).ab,ti.
124	(median adj neuropath*).ab,ti.
123	(rotator cuff and (injur* or disorder*)).ab,ti.
122	tennis elbow.ab,ti.
121	"thoracic outlet syndrome*".ab,ti.
120	(elbow* and (pain* or sprain* or strain* or injur* or impair*)).ab,ti.
119	(hand* and (pain* or sprain* or strain* or injur* or impair*)).ab,ti.
118	(wrist* and (pain* or sprain* or strain* or injur* or impair*)).ab,ti.
117	(arm* and (pain* or sprain* or strain* or injur* or impair*)).ab,ti.
116	(forearm* and (pain* or sprain* or strain* or injur* or impair*)).ab,ti.
115	(shoulder and capsul* and (sprain* or tear*)).ab,ti.
114	frozen shoulder.ab,ti.
113	painful arc.ab,ti.
112	biceps tend?nitis.ab,ti.
111	((supraspinatus or infraspinatus or subscapularis or teres minor or teres major or trapezius or deltoid or bicep* or bicipital or coracobrachialis) and (impingement or strain* or tear* or pain*)).ab,ti.
110	(rotator cuff and (sprain* or strain* or tear* or bursitis tendinitis or impingement)).ab,ti.
109	((glenohumeral or scapul* or acromioclavicular) and (pain* or sprain* or strain* or injur*)).ab,ti.
108	(shoulder* and (tendinopathy or tendinitis or tendonitis or capsulitis)).ab,ti.
107	(shoulder* and (pain* or sprain* or strain* or injur* or impair* or impingement)).ab,ti.
106	(lateral and (epicondylitis or epicondylosis or epicondylopathy)).ab,ti.
105	(medial and (epicondylitis or epicondylosis or epicondylopathy)).ab,ti.
104	carpal tunnel syndrome.ab,ti.
103	Thoracic Outlet Syndrome/
102	Bursitis/
101	exp Ulnar Neuropathies/
100	Radial Neuropathy/
99	exp Tendinopathy/
98	Finger Injuries/
97	Wrist Injuries/
96	exp Hand Injuries/
95	exp Arm Injuries/
94	Shoulder/in [Injuries]
93	Shoulder Joint/in [Injuries]
92	Shoulder Impingement Syndrome/
91	exp Median Neuropathy/
90	exp Cumulative Trauma Disorders/
89	Shoulder Pain/
88	or/44-87
87	"vertebrogenic adj3 pain*".ab,ti.
86	"tailbone adj3 pain*".ab,ti.
85	spondylosis.ab,ti.
84	(spinal adj2 stenos?s).ab,ti.
83	(SI adj2 joint).ab,ti.
82	"sciatic*".ab,ti.
81	(sacroiliac or sacro-iliac).ab,ti.
80	(sacrococcygeal adj2 pain*).ab,ti.
79	(sacral adj2 pain*).ab,ti.
78	radiculalgia.ab,ti.
77	"Piriformis syndrome*".ab,ti.
76	"lumbosacr*".ab,ti.
75	lumboischialgia.ab,ti.
74	"lumbarsacr*".ab,ti.
73	(lumbar adj3 (pain or facet or nerve root* or osteoarthritis or radicul* or spinal stenosis or spondylo* or zygapophys*)).ab,ti.
72	"low*-back-pain*".ab,ti.
71	"low* back pain".ab,ti.
70	(lumbar disk* adj3 (extruded or degenerat* or herniat* or prolapse* or sequestered or slipped)).ab,ti.
69	(lumbar disc* adj3 (extruded or degenerat* or herniat* or prolapse* or sequestered or slipped)).ab,ti.
68	dorsalgia.ab,ti.
67	coccyx.ab,ti.
66	coccydynia.ab,ti.
65	(back pain or back-pain).ab,ti.
64	(backache* adj3 (injur* or pain*)).ab,ti.
63	(back adj3 (ache* or injur* or pain*)).ab,ti.
62	(avulsed lumbar adj3 (disc* or disk*)).ab,ti.
61	Spinal Stenosis/
60	Spinal Diseases/
59	Sciatica/
58	Sacrum/
57	Sacroiliac Joint/
56	Sacrococcygeal Region/
55	Polyradiculopathy/
54	Piriformis Muscle Syndrome/
53	Osteoarthritis, Spine/
52	Lumbosacral Region/in [Injuries]
51	exp Lumbosacral Plexus/
50	Lumbar Vertebrae/in [Injuries]
49	Intervertebral Disc Displacement/
48	Intervertebral Disc Degeneration/
47	Coccyx/in [Injuries]
46	exp Back Pain/
45	exp Back Injuries/
44	exp Back/
43	or/24-42
42	torticollis.ab,ti.
41	"brachial plexus neuropath*".ab,ti.
40	"radiculopath*".ab,ti.
39	"cervicodynia*".ab,ti.
38	"cervicalgia*".ab,ti.
37	"neckache*".ab,ti.
36	"neck ache*".ab,ti.
35	"cervical pain*".ab,ti.
34	"neck pain*".ab,ti.
33	"neck injur*".ab,ti.
32	whiplash.ab,ti.
31	Torticollis/
30	exp Brachial Plexus Neuropathies/
29	Radiculopathy/
28	exp Cervical Vertebrae/in [Injuries]
27	Neck Muscles/in [Injuries]
26	Neck Pain/
25	Neck Injuries/
24	Whiplash Injuries/
23	or/1-22
22	"Department of Veterans Affairs".ab,ti.
21	"Department of Defense".ab,ti.
20	submariner*.ab,ti.
19	soldier*.ab,ti.
18	sailor*.ab,ti.
17	((navy or navies or naval) adj3 (base* or facilit* or installation* or personnel or population* or service*)).ab,ti.
16	(military adj3 (base* or facilit* or installation* or personnel or population* or service*)).ab,ti.
15	marines.ab,ti.
14	marine corps.ab,ti.
13	marching.ab,ti.
12	limited-duty assignment*.ab,ti.
11	((defence or defense) adj3 (department* or force*)).ab,ti.
10	conscript*.ab,ti.
9	coast guard.ab,ti.
8	(army or armies).ab,ti.
7	armed forces.ab,ti.
6	air force*.ab,ti.
5	active duty.ab,ti.
4	Warfare/
3	United States Department of Veterans Affairs/
2	United States Department of Defense/
1	Military Personnel/

### CINAHL Search Stratey

Search run April 26, 2017 in CINAHL Plus with Full Text; 19 results with limiters ( English Language; Peer Reviewed; Exclude MEDLINE records)

Search run June 1, 2018 in CINAHL Plus with Full Text; 15 results

**Table:**

Search ID#	Search Terms
S163	S122 AND S140 AND S161
S162	S122 AND S140 AND S161
S161	S141 OR S142 OR S143 OR S144 OR S145 OR S146 OR S147 OR S148 OR S149 OR S150 OR S151 OR S152 OR S153 OR S154 OR S155 OR S156 OR S157 OR S158 OR S159 OR S160
S160	program* N3 (assess* or evaluat*)
S159	primary health care
S158	pathway* N3 (clinical or care)
S157	(integrated or interdisciplinary or interprofessional or multidisciplinary or multi-disciplinary) N3 (care or clinic or clinics or implement* or intervention* or military or model* or plan* or process* or program*or strateg* or system*)
S156	model* N1 care
S155	military N3 (care or clinic or clinics or hospital* or medical or medicine or program*)
S154	innovative N3 (intervention* or model* or plan* or process* or program*or strateg* or system*)
S153	implement* N3 (intervention* or model* or plan* or process* or program*or strateg* or system*)
S152	(health care or healthcare or health-care) N3 (clinic or clinics or delivery or implement* or intervention* or military or model* or plan* or process* or program*or services or strateg* or system* or team*)
S151	approach* N3 (collaborative or complementary or comprehensive or innovative or integrated)
S150	(MH "Patient Care Plans+")
S149	(MH “Military Nursing”)
S148	(MH "Military Medicine")
S147	(MH “Interprofessional Relations”)
S146	(MH "Integrative Medicine")
S145	(MH "Hospitals, Military")
S144	(MH "Health Services Administration")
S143	(MH "Health Promotion")
S142	(MH "Health Care Delivery, Integrated")
S141	(MH "Health Care Delivery")
S140	S123 OR S124 OR S125 OR S126 OR S127 OR S128 OR S129 OR S130 OR S131 OR S132 OR S133 OR S134 OR S135 OR S136 OR S137 OR S138 OR S139
S139	warfare
S138	submariner*
S137	soldier*
S136	sailor*
S135	(navy or navies or naval) N3 (base* or facilit* or installation* or personnel or population* or service*)
S134	military N3 (base* or facilit* or installation* or personnel or population* or service*)
S133	marine corps
S132	marching
S131	limited-duty assignment*
S130	(defence or defense) N3 (department* or force*)
S129	conscript*
S128	coast guard
S127	army or armies
S126	armed forces
S125	air force*
S124	active duty
S123	(MH "Military Personnel+")
S122	S58 OR S100 OR S118 OR S121
S121	S119 OR S120
S120	(musculoskeletal or musculo-skeletal or MSK) N3 (care or condition* or disabilit* or disorder* or injur* or pain or problem* or trouble*)
S119	(MH "Musculoskeletal Diseases")
S118	S101 OR S102 OR S103 OR S104 OR S105 OR S106 OR S107 OR S108 OR S109 OR S110 OR S111 OR S112 OR S113 OR S114 OR S115 OR S116 OR S117
S117	plantar fasciitis
S116	tendinopathy
S115	tendinosis
S114	patellofemoral pain syndrome*
S113	toe* N3 (injur* or pain* or turf)
S112	thigh* N3 (injur* or pain*)
S111	leg* N3 (injur* or pain*)
S110	knee* N3 (injur* or pain*)
S109	hip* N3 (injur* or pain*)
S108	foot N3 (injur* or pain*)
S107	buttock* N3 (injur* or pain*)
S106	(talofibular or calcaneofibular or calcaneotibial or tibio*) N3 (sprain* or strain* or injur*)
S105	ankle* N3 (sprain* or strain* or injur*)
S104	lower N3 (extremit* or limb* or injur*)
S103	(MH "Plantar Fasciitis")
S102	(MH "Leg Injuries+")
S101	(MH "Hip Injuries+")
S100	S59 OR S60 OR S61 OR S62 OR S63 OR S64 OR S65 OR S66 OR S67 OR S68 OR S69 OR S70 OR S71 OR S72 OR S73 OR S74 OR S75 OR S76 OR S77 OR S78 OR S79 OR S80 OR S81 OR S82 OR S83 OR S84 OR S85 OR S86 OR S87 OR S88 OR S89 OR S90 OR S91 OR S92 OR S93 OR S94 OR S95 OR S96 OR S97 OR S98 OR S99
S99	repetit* N3 (strain* or sprain* or injur* or disorder*)
S98	cumulative trauma disorder*
S97	(radial or ulnar) N3 neuropath*
S96	upper extremit* injur*
S95	bursitis
S94	radial N3 neuropath*
S93	median N3 neuropath*
S92	rotator cuff N3 (injur* or disorder*)
S91	tennis elbow
S90	thoracic outlet syndrome*
S89	elbow* N3 (pain* or sprain* or strain* or injur* or impair*)).
S88	hand* N3 (pain* or sprain* or strain* or injur* or impair*)
S87	wrist* N3 (pain* or sprain* or strain* or injur* or impair*)
S86	(arm* N3 (pain* or sprain* or strain* or injur* or impair*)
S85	forearm* N3 (pain* or sprain* or strain* or injur* or impair*)
S84	shoulder and capsul* N3 (sprain* or tear*)
S83	frozen shoulder
S82	painful arc
S81	biceps tend?nitis
S80	(supraspinatus or infraspinatus or subscapularis or teres minor or teres major or trapezius or deltoid or bicep* or bicipital or coracobrachialis) N3 (impingement or strain* or tear* or pain*)
S79	rotator cuff N3 (sprain* or strain* or tear* or bursitis tendinitis or impingement)
S78	(glenohumeral or scapul* or acromioclavicular) N3 (pain* or sprain* or strain* or injur*)
S77	shoulder* N3 (tendinopathy or tendinitis or tendonitis or capsulitis)
S76	shoulder* N3 (pain* or sprain* or strain* or injur* or impair* or impingement)
S75	lateral N3 (epicondylitis or epicondylosis or epicondylopathy)
S74	medial N3 (epicondylitis or epicondylosis or epicondylopathy)
S73	carpal tunnel syndrome
S72	(MH "Thoracic Outlet Syndrome")
S71	(MH "Carpal Tunnel Syndrome")
S70	(MH "Bursitis")
S69	(MH "Ulnar Neuropathies+")
S68	(MH "Tendinopathy")
S67	(MH "Finger Injuries+")
S66	(MH "Wrist Injuries+")
S65	(MH "Hand Injuries+")
S64	(MH "Arm Injuries+")
S63	(MH "Shoulder/IN")
S62	(MH "Shoulder Joint/IN")
S61	(MH "Shoulder Impingement Syndrome")
S60	(MH "Cumulative Trauma Disorders+")
S59	(MH "Shoulder Pain")
S58	S18 OR S57
S57	S19 OR S20 OR S21 OR S22 OR S23 OR S24 OR S25 OR S26 OR S27 OR S28 OR S29 OR S30 OR S31 OR S32 OR S33 OR S34 OR S35 OR S36 OR S37 OR S38 OR S39 OR S40 OR S41 OR S42 OR S43 OR S44 OR S45 OR S46 OR S47 OR S48 OR S49 OR S50 OR S51 OR S52 OR S53 OR S54 OR S55 OR S56
S56	vertebrogenic N3 pain*
S55	tailbone N3 pain*
S54	spondylosis
S53	spinal stenos?s
S52	SI N2 joint
S51	sciatic*
S50	sacroiliac or sacro-iliac
S49	sacrococcygeal N3 pain*
S48	sacral N3 pain*
S47	radiculalgia
S46	Piriformis syndrome*
S45	lumbosacr*
S44	lumboischialgia
S43	lumbarsacr*
S42	lumbar N3 (pain or facet or nerve root* or osteoarthritis or radicul* or spinal stenosis or spondylo* or zygapophys*)
S41	low*-back-pain*
S40	low* back pain*
S39	lumbar disk* N3 (extruded or degenerat* or herniat* or prolapse* or sequestered or slipped)
S38	lumbar disc* N3 (extruded or degenerat* or herniat* or prolapse* or sequestered or slipped)
S37	dorsalgia
S36	coccy*
S35	back-pain
S34	backache* N3 (injur* or pain*)
S33	back N3 (ache* or injur* or pain*)
S32	avulsed lumbar N3 (disc* or disk*)
S31	(MH "Spinal Stenosis")
S30	(MH "Sciatica")
S29	(MH "Sacrum")
S28	(MH "Sacroiliac Joint")
S27	(MH "Polyradiculopathy+")
S26	(MH "Piriformis Muscles")
S25	(MH "Osteoarthritis, Spine+")
S24	(MH "Lumbosacral Plexus")
S23	(MH "Lumbar Vertebrae/IN")
S22	(MH "Intervertebral Disk Displacement")
S21	(MH "Coccyx/IN")
S20	(MH "Back Injuries+")
S19	(MH "Back Pain") OR (MH "Low Back Pain")
S18	S1 OR S2 OR S3 OR S4 OR S5 OR S6 OR S7 OR S8 OR S9 OR S10 OR S11 OR S12 OR S13 OR S14 OR S15 OR S16 OR S17
S17	torticollis
S16	brachial plexus neuropath*
S15	radiculopath*
S14	cervicodynia*
S13	cervicalgia*
S12	neck n1 ache* OR neckache*
S11	neck n3 pain*
S10	neck n3 injur*
S9	whiplash
S8	(MH "Torticollis")
S7	(MH "Brachial Plexus Neuropathies")
S6	(MH "Radiculopathy")
S5	(MH "Cervical Vertebrae/IN")
S4	(MH "Neck Muscles/IN")
S3	(MH "Neck Injuries")
S2	(MH "Neck Pain")
S1	(MH "Whiplash Injuries")

### Embase Search Strategy

Search run April 25, 2017 in Embase Classic+Embase 1947 to 2017 April 24; 883 results

Search run June 1, 2018 in Embase Classic+Embase 1947 to 2018 May 31; 96 results

**Table:**

# ▲	Searches
1	soldier/
2	warfare/
3	active duty.ab,ti.
4	air force*.ab,ti.
5	armed forces.ab,ti.
6	(army or armies).ab,ti.
7	coast guard.ab,ti.
8	conscript*.ab,ti.
9	((defence or defense) adj3 (department* or force*)).ab,ti.
10	limited-duty assignment*.ab,ti.
11	marching.ab,ti.
12	marine corps.ab,ti.
13	marines.ab,ti.
14	(military adj3 (base* or facilit* or installation* or personnel or population* or service*)).ab,ti.
15	((navy or navies or naval) adj3 (base* or facilit* or installation* or personnel or population* or service*)).ab,ti.
16	sailor*.ab,ti.
17	soldier*.ab,ti.
18	submariner*.ab,ti.
19	or/1-18
20	whiplash injury/
21	neck injury/
22	neck pain/
23	neck muscle/
24	exp cervical spine/
25	radiculopathy/
26	brachial plexus neuropathy/
27	torticollis/
28	whiplash.ab,ti.
29	"neck injur*".ab,ti.
30	"neck pain*".ab,ti.
31	"neck ache*".ab,ti.
32	"neckache*".ab,ti.
33	"brachial plexus neuropath*".ab,ti.
34	torticollis.ab,ti.
35	or/20-34
36	backache/
37	coccygeal bone/
38	intervertebral disc degeneration/
39	intervertebral disk hernia/
40	lumbar vertebra/
41	spondylosis/
42	ischialgia/
43	sacrum/
44	spine disease/
45	vertebral canal stenosis/
46	(back adj3 (ache* or injur* or pain*)).ab,ti.
47	(backache* adj3 (injur* or pain*)).ab,ti.
48	(back pain or back-pain).ab,ti.
49	(lumbar disc* adj3 (extruded or degenerat* or herniat* or prolapse* or sequestered or slipped)).ab,ti.
50	(lumbar disk* adj3 (extruded or degenerat* or herniat* or prolapse* or sequestered or slipped)).ab,ti.
51	"low* back pain".ab,ti.
52	(lumbar adj3 (pain or facet or nerve root* or osteoarthritis or radicul* or spinal stenosis or spondylo* or zygapophys*)).ab,ti.
53	"Piriformis syndrome*".ab,ti.
54	(sacral adj2 pain*).ab,ti.
55	((spine or spinal) adj4 (condition* or disable* or disabilit* or disorder* or pain or stenos?s)).ab,ti.
56	spondylosis.ab,ti.
57	or/36-56
58	shoulder pain/
59	cumulative trauma disorders/
60	median neuropathy/
61	shoulder impingement syndrome/
62	exp arm injuries/
63	exp hand injuries/
64	exp tendinopathy/
65	radial neuropathy/
66	exp ulnar neuropathies/
67	bursitis/
68	thoracic outlet syndrome/
69	carpal tunnel syndrome.ab,ti.
70	(medial and (epicondylitis or epicondylosis or epicondylopathy)).ab,ti.
71	(lateral and (epicondylitis or epicondylosis or epicondylopathy)).ab,ti.
72	(shoulder* and (pain* or sprain* or strain* or injur* or impair* or impingement)).ab,ti.
73	(shoulder* and (tendinopathy or tendinitis or tendonitis or capsulitis)).ab,ti.
74	((glenohumeral or scapul* or acromioclavicular) and (pain* or sprain* or strain* or injur*)).ab,ti.
75	(rotator cuff and (sprain* or strain* or tear* or bursitis tendinitis or impingement)).ab,ti.
76	((supraspinatus or infraspinatus or subscapularis or teres minor or teres major or trapezius or deltoid or bicep* or bicipital or coracobrachialis) and (impingement or strain* or tear* or pain*)).ab,ti.
77	biceps tend?nitis.ab,ti.
78	painful arc.ab,ti.
79	frozen shoulder.ab,ti.
80	(shoulder and capsul* and (sprain* or tear*)).ab,ti.
81	(forearm* and (pain* or sprain* or strain* or injur* or impair*)).ab,ti.
82	(arm* and (pain* or sprain* or strain* or injur* or impair*)).ab,ti.
83	(wrist* and (pain* or sprain* or strain* or injur* or impair*)).ab,ti.
84	(hand* and (pain* or sprain* or strain* or injur* or impair*)).ab,ti.
85	(elbow* and (pain* or sprain* or strain* or injur* or impair*)).ab,ti.
86	"thoracic outlet syndrome*".ab,ti.
87	tennis elbow.ab,ti.
88	(rotator cuff and (injur* or disorder*)).ab,ti.
89	(median adj neuropath*).ab,ti.
90	(radial adj neuropath*).ab,ti.
91	bursitis.ab,ti.
92	"upper extremit* injur*".ab,ti.
93	((radial or ulnar) adj neuropath*).ab,ti.
94	"cumulative trauma disorder*".ab,ti.
95	(repetit* and (strain* or sprain* or injur* or disorder*)).ab,ti.
96	or/58-95
97	exp hip injury/
98	exp leg injury/
99	knee ligament/
100	exp foot injury/
101	exp ankle injury/
102	exp collateral ligaments/
103	plantar fasciitis/
104	(lower and (extremit* or limb* or injur*)).ab,ti.
105	(ankle* and (sprain* or strain* or injur*)).ab,ti.
106	((talofibular or calcaneofibular or calcaneotibial or tibio*) and (sprain* or strain* or injur*)).ab,ti.
107	(buttock* and (injur* or pain*)).ab,ti.
108	(foot and (injur* or pain*)).ab,ti.
109	(hip* and (injur* or pain*)).ab,ti.
110	(knee* and (injur* or pain*)).ab,ti.
111	(leg* and (injur* or pain*)).ab,ti.
112	(thigh* and (injur* or pain*)).ab,ti.
113	(toe* and (injur* or pain* or turf)).ab,ti.
114	"patellofemoral pain syndrome*".ab,ti.
115	tendinosis.ab,ti.
116	tendinopathy.ab,ti.
117	plantar fasciitis.ab,ti.
118	or/97-117
119	musculoskeletal disease/
120	((musculoskeletal or musculo-skeletal or MSK) adj4 (care or condition* or disabilit* or disorder* or injur* or pain or problem* or trouble*)).ab,ti.
121	119 or 120
122	118 or 121
123	health care delivery/
124	integrated health care system/
125	health care planning/
126	health promotion/
127	integrative medicine/
128	military medicine/
129	(approach* adj3 (collaborative or complementary or comprehensive or innovative or integrated)).ab,ti.
130	barrier*.ab,ti.
131	facilitator*.ab,ti.
132	((health care or healthcare or health-care) adj3 (clinic or clinics or delivery or implement* or intervention* or military or model* or plan* or process* or program*or services or strateg* or system* or team*)).ab,ti.
133	(implement* adj3 (intervention* or model* or plan* or process* or program*or strateg* or system*)).ab,ti.
134	(innovative adj3 (intervention* or model* or plan* or process* or program*or strateg* or system*)).ab,ti.
135	(military adj3 (care or clinic or clinics or hospital* or medical or medicine or program*)).ab,ti.
136	(model* adj care).ab,ti.
137	((integrated or interdisciplinary or interprofessional or multidisciplinary or multi-disciplinary) adj3 (care or clinic or clinics or implement* or intervention* or military or model* or plan* or process* or program*or strateg* or system*)).ab,ti.
138	(pathway* adj3 (clinical or care)).ab,ti.
139	primary health care.ab,ti.
140	(program* adj3 (assess* or evaluat*)).ab,ti.
141	or/123-140
142	19 and (35 or 57 or 96 or 122) and 141
143	limit 142 to english language
144	limit 143 to (conference abstract or conference paper or "conference review" or editorial or letter)
145	143 not 144

## Abbreviations

COM-B: Capability, opportunity, motivation-behavior
EPOC: Cochrane Effective Practice and Organization of Care
PRESS: Peer Review of Electronic Search Strategies
PRISMA: Preferred Reporting Items for Systematic Reviews and Meta-analyses
PRISMA-ScR: Preferred Reporting Items for Systematic Reviews and Meta-analyses extension for Scoping Reviews
US: United States
